# Integration of high-throughput phenotyping with anatomical traits of leaves to help understanding lettuce acclimation to a changing environment

**DOI:** 10.1007/s00425-022-03984-2

**Published:** 2022-09-02

**Authors:** Chiara Amitrano, Astrid Junker, Nunzio D’Agostino, Stefania De Pascale, Veronica De Micco

**Affiliations:** 1 Department of Agricultural Sciences, University of Naples Federico II, Portici, NA Italy; 2 Leibniz Institute of Plant Genetics and Crop Plant Research, OT Gatersleben, Corrensstr. 3, 06466 Seeland, Germany

**Keywords:** Vapor pressure deficit, Leaf anatomy, Plant hydraulics, Photosynthesis, Short-term acclimation, Stomatal traits

## Abstract

**Main conclusion:**

The combination of image-based phenotyping with in-depth anatomical analysis allows for a thorough investigation of plant physiological plasticity in acclimation, which is driven by environmental conditions and mediated by anatomical traits.

**Abstract:**

Understanding the ability of plants to respond to fluctuations in environmental conditions is critical to addressing climate change and unlocking the agricultural potential of crops both indoor and in the field. Recent studies have revealed that the degree of eco-physiological acclimation depends on leaf anatomical traits, which show stress-induced alterations during organogenesis. Indeed, it is still a matter of debate whether plant anatomy is the bottleneck for optimal plant physiology or vice versa. Here, we cultivated ‘Salanova’ lettuces in a phenotyping chamber under two different vapor pressure deficits (VPDs; low, high) and watering levels (well-watered, low-watered); then, plants underwent short-term changes in VPD. We aimed to combine high-throughput phenotyping with leaf anatomical analysis to evaluate their capability in detecting the early stress signals in lettuces and to highlight the different degrees of plants’ eco-physiological acclimation to the change in VPD, as influenced by anatomical traits. The results demonstrate that well-watered plants under low VPD developed a morpho-anatomical structure in terms of mesophyll organization, stomatal and vein density, which more efficiently guided the acclimation to sudden changes in environmental conditions and which was not detected by image-based phenotyping alone. Therefore, we emphasized the need to complement high-throughput phenotyping with anatomical trait analysis to unveil crop acclimation mechanisms and predict possible physiological behaviors after sudden environmental fluctuations due to climate changes.

**Supplementary Information:**

The online version contains supplementary material available at 10.1007/s00425-022-03984-2.

## Introduction

It is estimated that nowadays about 50% of the global crop yield loss is due to climate change which has increased temperature, severity, and frequency of drought, also causing long periods of severe air vapor pressure deficit (VPD), threatening the sustainability of future agriculture (IPCC 2017).

Furthermore, climate change imposes sudden fluctuations in environmental conditions (Cramer et al. 2011; Will et al. 2013) which threaten agricultural production, affecting crop yield, biomass, biochemical composition, and visual quality of plants, thus impacting the marketability of products (Rouphael et al. 2018).

Plants react to environmental changes by implementing a series of morpho-physiological strategies, the extent of which depends on the plasticity of the species in acclimation (Amitrano et al. 2021a; Choat et al. 2018). When subjected to any environmental stress, plant cells activate a signaling cascade, including hormones, secondary messengers, and signal transducers, which converge to regulate the expression of genes that encode for proteins and enzymes involved in the stress response. This leads to a biochemical and physiological reprogramming, e.g., the accumulation of reactive oxygen species (ROS) or the closure of stomata (Zandalinas et al. 2018).

For example, since most crops perceive high air VPD as an “atmospheric water stress” (Vadez et al. 2013; Will et al. 2013), they try to react by modifying stomatal regulation, with consequences on photosynthesis, to maintain the water balance (Amitrano et al. 2021b). However, while adjusting their physiological processes, plants cannot exceed the limits imposed by their anatomical structure (stomatal size and density, organization of the mesophyll, vein density, etc.), which has, therefore, been hypothesized to be a bottleneck for optimal functioning (Korner 2015). Indeed, due to its ability to influence plant tissue development, VPD has been recognized as the “hidden driver” behind morpho-physiological traits of plants growing in controlled environment (Amitrano et al. 2019). For instance, small leaves are more common in dry environments (under high VPD), as plants can react to adverse conditions by reducing transpiration costs (Sack and Scoffoni 2013), and where turgor-driven cell enlargement is reduced. Indeed, the thickness of the boundary layer increases with the size of the leaf, so it would be difficult for larger leaves to reduce the heat loss in a dry environment (Wang et al. 2020). Therefore, the evaporative demand (expressed as VPD) and the water availability to which the plants are subjected are closely linked, influencing not only the entire plant–water relationships, but also the size of the leaves and other morpho-anatomical traits (Scoffoni et al. 2015). For these reasons, the plasticity of water-related leaf traits, such as the ability to limit transpiration in conditions of high VPD, is promising to alleviate the stress, while allowing water saving, and increasing yield under limiting environmental conditions (Wang et al. 2020). Furthermore, narrower leaves allow to save “construction costs” attributed to more expanded vascular and cell-wall fractions, with a competitive advantage in harsh environments, where the availability of carbon and water is already insufficient (Carins Murphy et al. 2012; Niinemets et al. 2007). The morpho-anatomical traits of plants, measurable at the level of single organism (Kattge et al. 2020), depend on the genetic properties of the species and reflect their evolutionary lineage, but are strongly influenced by the surrounding environment (Valladares 2007; Violle et al. 2007). Recently, plant traits have been used in ecology studies and vegetation modelling as they can act as a proxy to predict how plants behave when subjected to different environmental stimuli, how they will affect other trophic levels, and how they will influence the whole ecosystem (Garnier and Navas 2012; Wright et al. 2017). In a climate change scenario, crop research focused on plant traits is becoming essential to achieve the optimization of resource use and maximization of production, ultimately ensuring food security and the livelihood of a growing population, also thanks to technological innovation in agriculture (Ray et al. 2013; Rockström et al. 2009). In this motivating context, high-throughput phenotyping technologies have been developed to characterize plant responses using image analysis in a non-destructive and non-invasive way (Furbank and Tester 2011; Tardieu et al. 2017), and to highlight the coordination of morpho-physiological and metabolic traits (Poorter et al. 2009). The response to abiotic stresses, including water shortage, heat, and salt stress, has been extensively studied in crops using phenotyping platforms that quantitatively analyze geometric-, color-, fluorescence- and NIR-related traits (Dobrowski et al. 2005; Woo et al. 2008; Mehta et al. 2010; Klukas et al. 2014). As an example, Kim et al. (2020) used image-based phenotyping to determine the water use efficiency, water loss rate and transpiration rate of rice cultivars under drought stress and to detect the difference between tolerant and susceptible plants. However, there is still a gap between the whole-plant phenotyping level and the molecular and anatomical level (Costa et al. 2019; Langstroff et al. 2021), therefore, as many traits as possible should be collected to integrate phenotypic data points and facilitate the integration of knowledge also through modelling (Van Eeuwijk et al. 2019).

To the best of our knowledge, no studies have attempted to comprehensively correlate the effects of VPD and water stress on morpho-anatomical and physiological development of crops, considering the combination of high-throughput phenotyping with observation at microscopic level. Therefore, this study proposes the integration of automated image-based phenotyping with in-depth anatomical analysis of plant tissue to detect the early stress symptoms of plants subjected to different environmental conditions and to investigate the anatomy-driven mechanisms of plant plasticity in acclimation, highlighting the structure-to-function relationships. We aimed to evaluate whether the analysis of anatomical traits can represent an added value to high-throughput phenotyping allowing to predict the degree of eco-physiological acclimation of plants to sudden changes in VPD, as influenced by anatomical traits.

Indeed, establishing the appropriate combination of air and water supply would depend on the environmental conditions, such as radiation, temperature, and relative humidity, and it is essential for maintaining the desired photosynthetic performance. To this end, we cultivated green and red ‘Salanova’ lettuce (*Lactuca sativa* L. var. *capitata*) plants in a phenotyping growth chamber in two cycles with low and high VPD and two irrigation levels (well-watered and low-watered) and then subjected them to short-term changes in the VPD condition. The results obtained provided useful information to understand how to improve the performance of lettuce which is the most common vegetable cultivated in controlled environment (Inoue et al. 2021) and could be a starting point for extending the results to field crops to model their responses when subjected to sudden weather fluctuations.

## Materials and methods

### Plant material and experimental design

Seeds of green and red ‘Salanova’ lettuce (*Lactuca sativa* L. var. *capitata*) were provided by Rijk Zwaan (Rijk Zwaan, Der Lier, The Netherlands). For each trial, 80 green and 80 red ‘Salanova’ seeds were germinated in 10 cm pots on soil (red substrate 2, Klasmann–Deilmann GmbH, Geeste, Germany) in a walk-in growth-chamber at the IPK Leibniz‐Institute of Plant Genetics and Crop Plant Research in Gatersleben (Germany) under controlled conditions (23/19 °C day/night, 75% RH, 315 µmol photons m^−2^ s^−1^, 12 h photoperiod). Light was provided by halide lamps (Venture Lighting Europe Ltd., Rickmansworth, Hertfordshire, England).

**Fig. 1:**
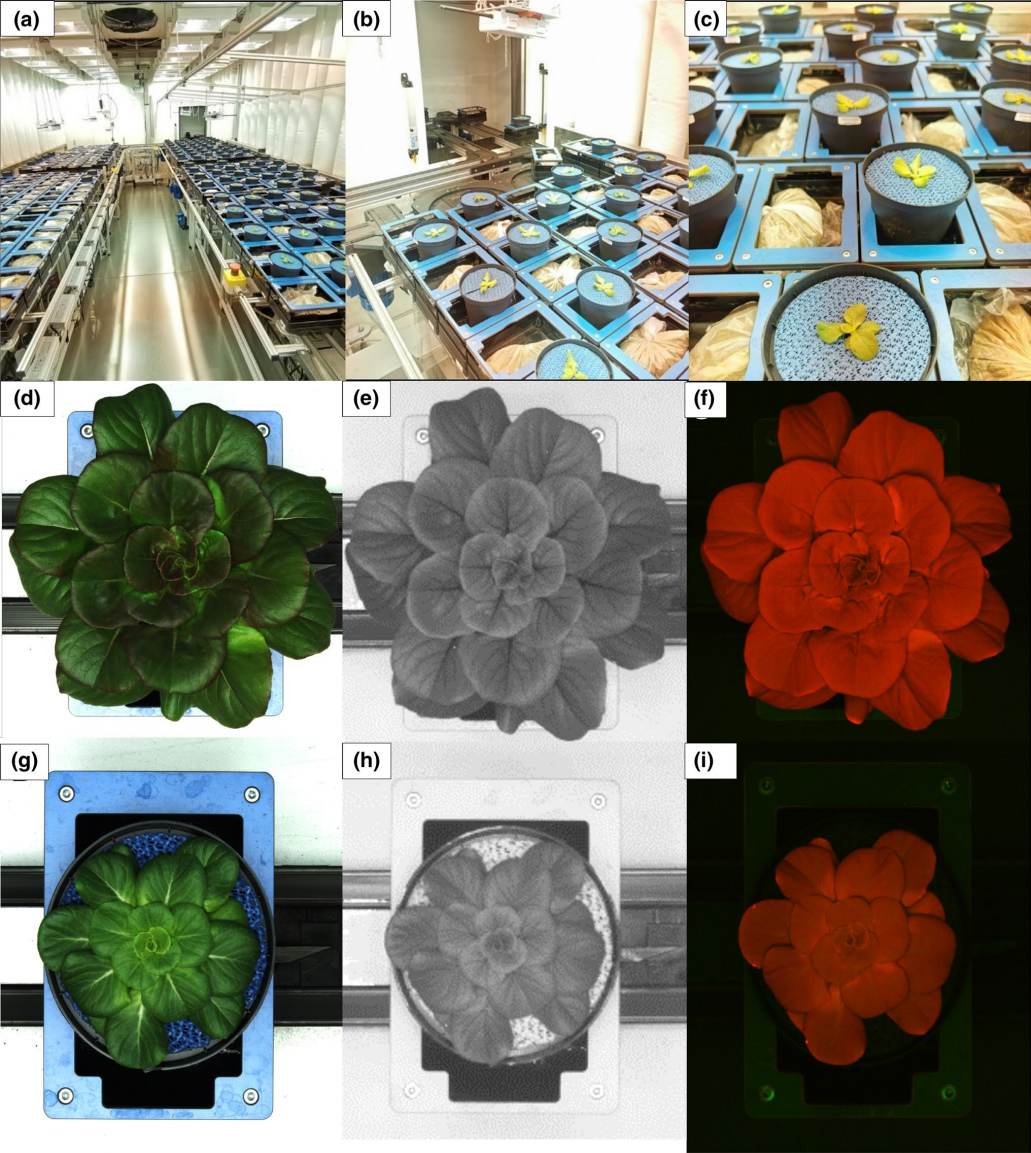
Green and red ‘Salanova’ plants in the LemnaTec carriers of the small-plant phenotyping chamber (PGC) at IPK–Gatersleben. The surface of pots was covered with a blue rubber mat (**a**–**c**). The phenotype of a representative plant under WW (**d**–**f**) and WL (**g**–**i**) conditions: images acquired with the three different sensors of the cameras used in the HT automated screening system for small plants: imaging in the visible light spectrum (VIS) (**d**, **g**), fluorescence imaging (FLUO) (**e**, **h**), and NIR light spectrum imaging (**f**, **i**)

After 10 days, the more uniform 64 green and 64 red plants at the stage of 4 true leaves were moved to the Phenotyping Growth Chamber (PGC) and placed in the LemnaTec carriers (Fig. 1a–c). To prevent evaporation from the soil and to provide a uniform background for the phenotyping cameras, the soil surface of all pots was covered with a blue rubber mat (Fig. 1a–c). A few days before the experiments, the soil water content corresponding to 100% of field capacity (FC) was determined by weighing soil-filled pots after full watering and after drying for 3 days at 80 °C as reported in Junker et al. (2015).

Once in the PGC, half of the plants were irrigated at 100% FC and were referred to as WW (well-watered) and the other half at 30% FC and were referred to as LW (low-watered). The irrigation took place via an automatic weighing/watering station with a pump that irrigates the lower container, to avoid any interference with the growth of the lettuce.

**Fig. 2:**
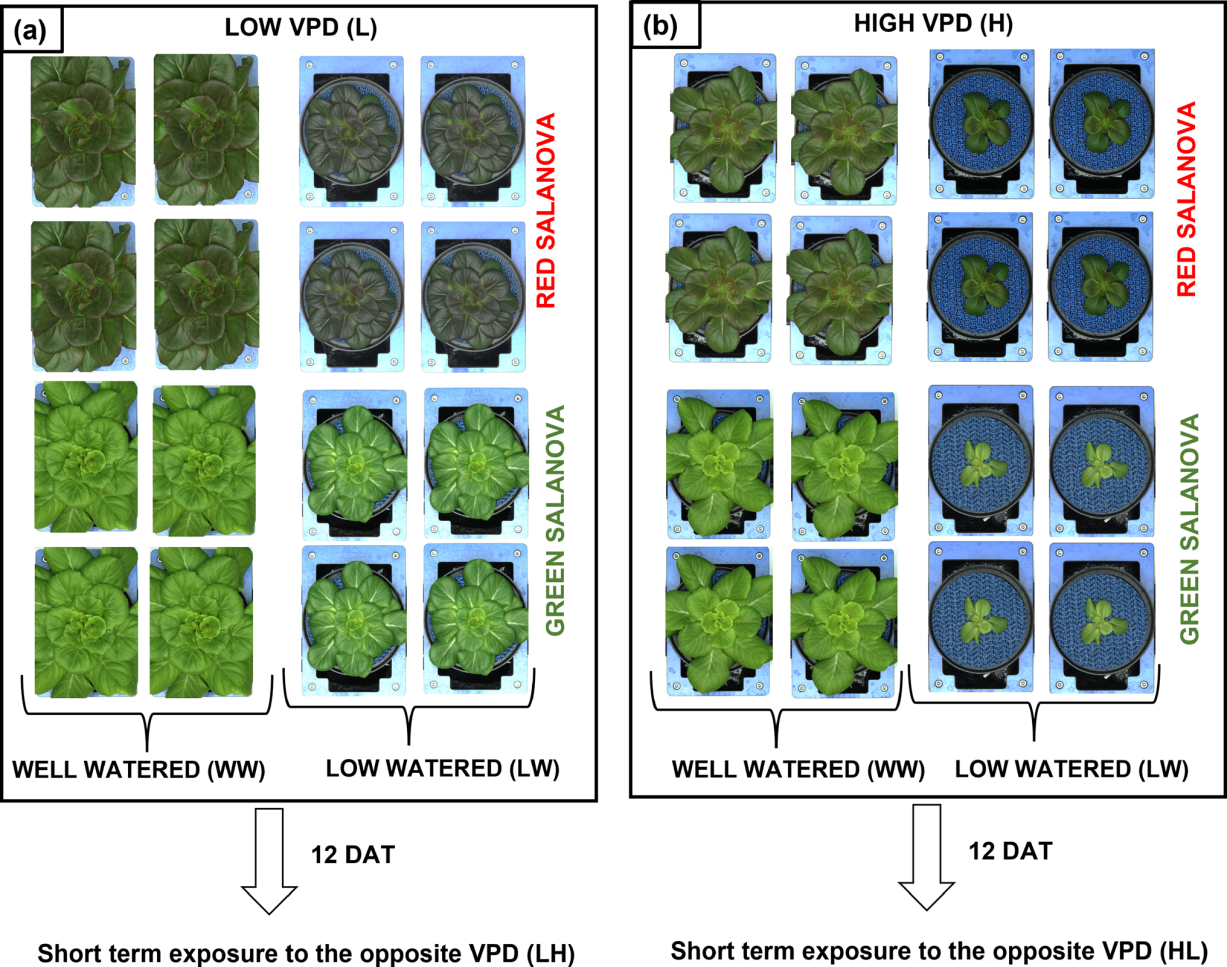
Summary of the experimental design showing green (G) and red (R) ‘Salanova’ plants irrigated with the two watering levels (well-watered, WW; low-watered, LW), grown in the phenotyping chamber in the two consecutive cycles under different VPDs: low VPD (**a**), high VPD (**b**). Short-term exposure under the opposite VPD is also represented

Two experimental trials were subsequently conducted in the PGC at 2 different environmental VPDs. VPD values were obtained by keeping temperature fixed and accordingly modifying the relative humidity (RH %) as reported in Amitrano et al. (2021a, b): (i) the first trial was carried out at a VPD of 0.7 kPa (Low VPD; L); (ii) the second one at 1.4 kPa (High VPD; H). Average values and amplitude of VPD levels were decided according to the values typically monitored in plant factories and greenhouses (Garcia et al. 2011; Lu et al. 2015; Zhang et al. 2015). Twelve days after the transferring (DAT) of plants in the PGC, the environmental conditions in the chamber were changed and plants were kept for 5 days at the opposite VPD (from Low to High, LH; and from High to Low, LH) (Fig. 2) to simulate a short term exposure to the opposite VPD and test their short-term acclimation ability, following the approach reported in Amitrano et al. (2021a, b). These experiments focused on lettuce plants at the beginning of the cultivation cycle, because we were interested to evidence possible plant sensitivity to changing environmental factors at an early stage of plant development.

### Image acquisition and analysis

All images were automatically captured using the LemnaTec system. All plants were imaged daily from the top view using three imaging procedures VIS, NIR, FLUO (Fig. 1d–i). VIS images were acquired in the visible light spectrum (~ 390–750 nm) using a Basler (Basler AG, Ahrensburg, Germany) Pilot piA2400-17gc (RGB) camera with a resolution of 2454 × 2056 pixels. A fluorescence imaging system (FLUO, excitation: 400–500 nm, emission: 520–750 nm) using a Basler Scout scA1400-17gc and (RGB) camera with a resolution of 1624 × 1234 pixels, allowed the quantification of static fluorescence signals of plants.

Near-infrared (NIR) imaging was performed in the wavelength range 1450–1550 nm using a Nir 300 PGE sensor (Allied Vision Technologies Gmb Hformer VDS Vosskühler GmbH, Stadtroda, Germany, monochrome camera) with a resolution of 320 × 254 pixels.

After transferring all plants to PGC, on days 12 and 22 (before and after the switch in environmental conditions), imaging of chlorophyll “a” fluorescence was performed using the FluorCam imaging fluorimeters (Photon Systems Instruments, Brno, Czech Republic).

Measurements of Φ_PSII_ were made after light adaptation of the plants in the adaptation tunnel and after a period of illumination in the FluorCam chamber as indicated in the “Results” section. The duration of the saturating light pulse to induce Fm was 800 ms with intensity of 4100 μmol m^−2^ s^−1^ (white light).

After 1 h of lights off, the dark-adapted plants were subjected to eight pulses of saturated light (800 ms; 4100 μmol m^−2^ s^−1^ white light) over the course of 145.8 s. After the first pulse of saturating light, the light was turned off (5 s) to measure *F*_0_, followed by the second pulse of saturating light and actinic light to measure maximum fluorescence (*F*_m_) followed by 10 s of dark relaxation to measure the Kautsky kinetics.

For the *F*_m_ quenching analysis, white light and actinic light were turned on for 120 s and supplemented by six saturating light pulses every 20 s followed by further 10 s dark relaxation measurements.

Image analysis was performed using the IAP (Integrated Analysis Platform) software 9 in different steps: image acquisition, pre-processing, feature extraction and post-processing. From the analysis of the images, the most important traits were extracted and classified as geometric (TA in the RGB channel), color-related (Y2G, Lab_a, Lab_b, R2G, hsv_h and hsv_v in the VIS channel and hsv_s in the FLUO channel), physiological-related (Int in the NIR channel) and (Fv/Fm, ΦPSII, NPQ in the chlorophyll fluorescence channel). All the above-mentioned measured traits and their biological meanings are summarized in Table S1 and reported in Tables 3 and 4 and in Figs. 1, 5 and 7.

### Biomass traits

Plants were harvested at 23 DAT and determination of fresh (FW) and dry (DW) weights of above-ground biomass was performed at the single plant level. After weighting the biomass for fresh weight, the dry biomass was determined after drying all the plants in oven at 80 °C for 72 h (until stable weight).

### Light microscopy and quantitative leaf anatomical traits

On harvest days (16 days after transferring into the PGC, still in the early stage of the cultivation cycle), a single fully expanded leaf from 6 plants per treatment was collected and fixed in F.A.A. solution (40% formaldehyde, glacial acetic acid, 50% ethanol, 5:5:90 by volume) and transferred to the PWA (plant and wood anatomy) laboratory of the University of Naples Federico II for light microscopy analysis. Each leaf was dissected to obtain sub-samples of about 5 × 5 mm in the median region thus avoiding any possible bias due to comparing portion of leaves with different position, age/degree of development. Samples were dehydrated in ethanol series up to 95% and then embedded in JB4 acrylic resin (Polysciences, Germany). Samples were cut into thin cross sections of about 5 µm by means of a rotating microtome and stained with 0.5% Toluidine blue in water, as reported in Feder and O’brien, (1968). Sections were observed under the BX51 light microscope (Olympus, Germany) and digital images were collected and analyzed using the Olympus EP50 digital camera and the Olympus CellSens 3.2 software to characterize the leaf lamina by measuring the thickness of upper (UET) and lower epidermis (LET) as well as the thickness of the palisade (PT) and spongy (ST) parenchyma, and the quantity of intercellular spaces, used as a proxy for the compactness of the parenchyma and expressed as percentage of tissue occupied by the intercellular spaces on a given surface (IS %). All measurements were made in the same three regions per cross section, taking care to avoid main veins, and then averaged to obtain a complete view of the traits under investigation.

Stomatal traits were determined on abaxial peels of the lamina, taken centrally in each leaflet, avoiding the main vein and the margin. Peels were analyzed under the above-mentioned microscope and five measurements from 3 different peels were performed.

Stomatal density (SD) was calculated as the number of stomata per mm^2^, measured with 20 × magnification, while the stomatal area (SA), expressed in µm^2^, was measured with 50 × magnification in 10 stomata per leaf, considering both the guard cell major (pole to pole) and minor axes to calculate the area of an imaginary ellipse, as reported in Amitrano et al. (2021b). Vein traits on the same leaves were also detected following the protocol by Sack and Scoffoni (2013).

Briefly, the leaves were chemically cleared in in series of dilutions with ethanol (up to 100%) and then double-stained using safranin (1% safranin in 100% ethanol) and fast green (1% fast green in 100% ethanol). Under light microscope, each leaf was imaged in three different areas and used to calculate the vein density (VD) as the ratio between the sum of the lengths of the veins of all orders and the area of the image (Amitrano et al. 2021a).

### Statistical analyses

To test the influence of the different independent factors: (i) VPD, (ii) cultivar (C), (iii) water (W) on dependent variables, a three-way analysis of variance (ANOVA) was performed with the IBM SPSS Statistics software (SPSS, Chicago, IL, USA). In case of significant interactions, the data were then subjected to one-way analysis of variance (ANOVA) and the mean values were separated according to Tukey’s test with *p* < 0.05. Line plots, correlation plots (*corrplot* package with the Spearman’s method) and the principal component analysis (PCA; *prcomp()* function) were all performed using the R software environment for statistical computing and graphics (version 4.4.1).

## Results

### Growth and biometry

At the end of the growth cycle, significant differences were found between ‘Salanova’ plants grown under low (L) and high (H) VPD at the two watering levels (well-watered, WW; low-watered, LW). As reported in Table 1, VPD (V), cultivar (C) and water level (W) had a significant effect alone and in combination (VPD × C × W) on plant biomass. Both fresh and dry weight followed the same trend with weight gains at low VPD in both WW and LW (L WW and L LW).

**Table 1:** Fresh (FW) and dry weight (DW) of above-ground biomass of ‘Salanova’ plants grown under Low (L) and High (H) VPD at the two watering levels (well-watered, WW; low-watered, LW)

	FW (g)	DW (g)
L G LW	4.12 ± 0.13^c^	0.45 ± 0.01^d^
L G WW	16.12 ± 0.53^a^	1.21 ± 0.03^a^
L R LW	3.96 ± 0.12^c^	0.39 ± 0.01^d^
L R WW	16.77 ± 0.38^a^	1.10 ± 0.02^b^
H G LW	1.58 ± 0.06^d^	0.21 ± 0.01^e^
H G WW	14.64 ± 0.34 ^b^	1.03 ± 0.03^c^
H R LW	2.00 ± 0.08^d^	0.21 ± 0.01^e^
H R WW	13.57 ± 0.44^b^	0.83 ± 0.08^c^
Significance		
VPD	***	***
C	**	**
W	***	***
VPD × C × W	***	***

Mean values and standard errors are reported. NS, *; **, and ***—not significant or significant at *p* < 0.05, 0.01, and 0.001, respectively. Different letters correspond to significantly different values (*p* < 0.05)

**Fig. 3:**
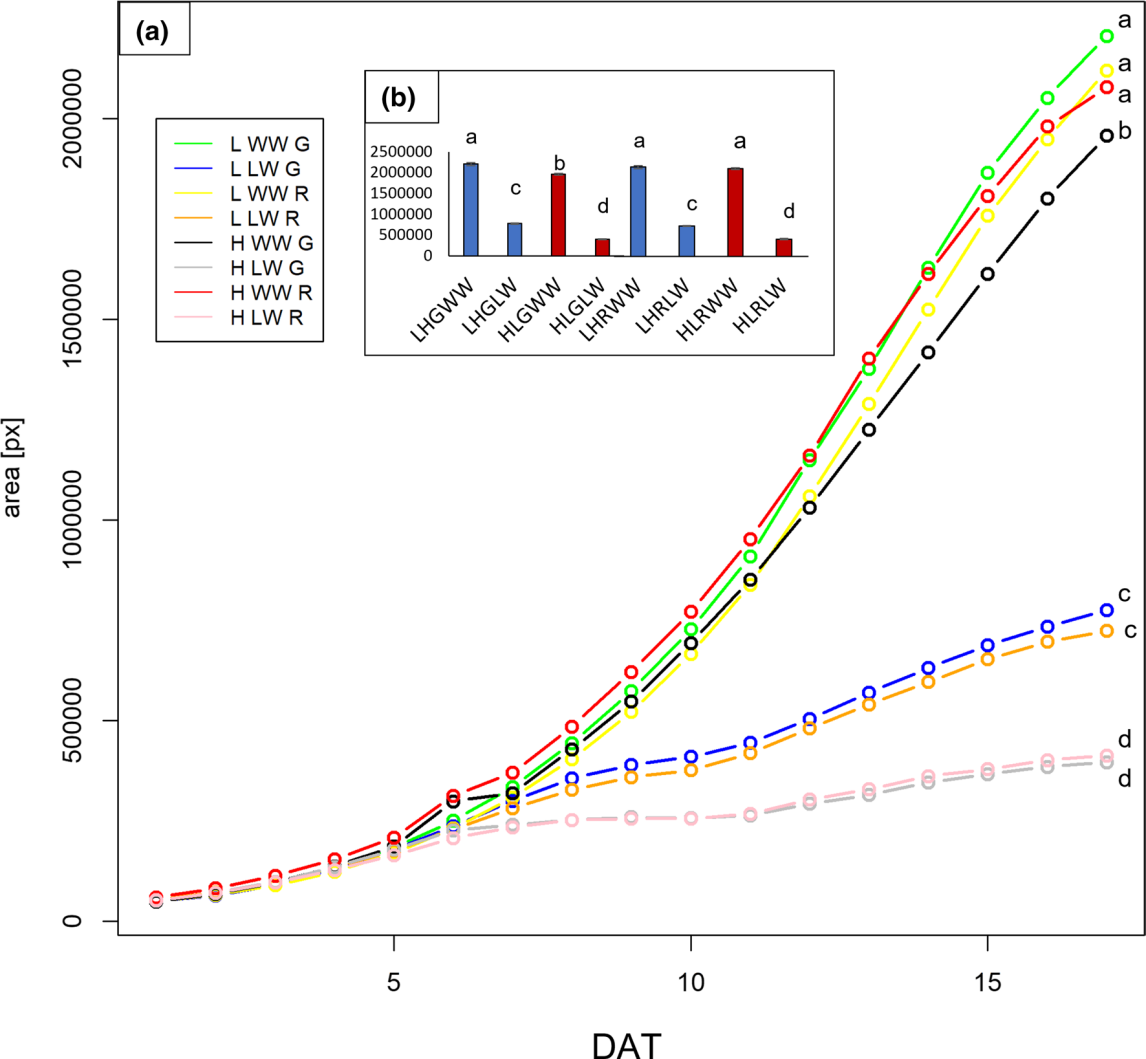
**a** Evolution of the growth of ‘Salanova’ plants in terms of total area during the entire cultivation period in the phenotyping chamber. **b** Area of the plants on the last day of cultivation after changing environmental conditions. Mean values are shown, for the end point different letters correspond to significantly different values between treatments per each DAT (*p* < 0.05)

In addition, both weights were enhanced in WW plants with increases of 66–77% compared with LW. No significant differences were found between green (G) and red (R) plants under WW and LW. In Fig. 3a, the growth in term of plant area expansion showed significant differences between the conditions.

Plant area increased at low VPD in both WW and LW watering levels. At 12 DAT the switch in environmental conditions only affected plants grown at LW, which underwent a slight bending, to promptly resume their exponential growth (Fig. 3b).

At the end of the growing period, only red plants under high VPD (H WW R) and then subjected to short-term low VPD exposure were able to reach values of plant area comparable with plants grown under low VPD (L WW G and R) but the same did not happen for plant subjected to low-watering levels.

### Quantitative anatomy

**Fig. 4:**
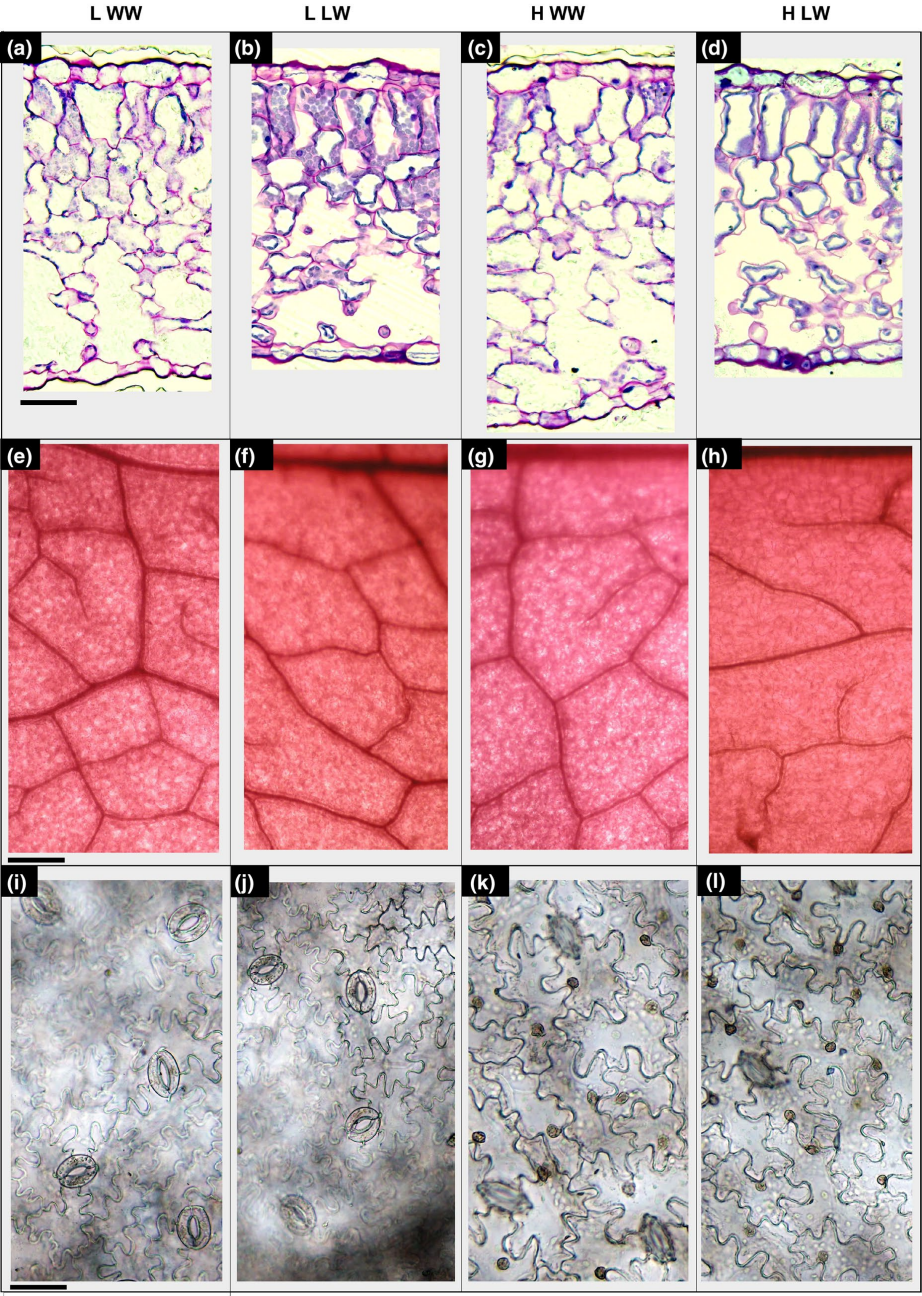
Light microscopy views of leaf lamina cross sections (**a**–**d**) and leaf epidermal peels showing venation (**e**–**h**) and stomata (**i**–**n**) randomly chosen among the many shots of ‘Salanova’ plants grown at: (i) low VPD and well-watered level (L WW; **a**, **e**, **i**), (ii) low VPD and low-watered level (L LW; **b**, **f**, **l**), (iii) high VPD and well-watered level (H WW; **c**, **g**, **m**), (iv) high VPD and low-watered level (H LW; **d**, **h**, **n**). The images in panel **a**, **b**, **c**, **d**, **i**, **l**, **m**, **n** are at the same magnification and scale bar is 50 µm; the images in panel **e**, **f**, **g**, **h** are at the same magnification and scale bar is 20 µm

Microscopy observations showed that the growth in the two VPD conditions and the two different watering levels did not affect the overall organization of the leaf tissues in qualitative terms. Indeed, as shown in Fig. 4, green and red plants under all the condition of VPD and watering levels maintained the typical dorsiventral structure, without any alterations in tissue organization due to the imposed environmental stress. However, the quantification of anatomical traits evidenced the occurrence of significant differences among the treatments. VPD (V), cultivar (C) and water level (W) had a significant effect alone and in combination (VPD × C × W) on the morpho-anatomical traits (Table 2).

**Table 2:** Leaf anatomical traits of green (G) and red (R) ‘Salanova’ plants grown under Low (L) and High (H) VPD at the two watering levels (well-watered, WW; low-watered, LW)

	UET (µm)	PT (µm)	ST (µm)	LET (µm)	LT (µm)	IS (%)	SD (n mm^−2^)	SA (µm^2^)	VD (mm mm^−2^)
L G LW	17.43 ± 0.62^a^	99.05 ± 2.31^b^	158.85 ± 1.36^b^	12.61 ± 0.36^b^	287.94 ± 4.65^b^	40.36 ± 1.89^bc^	55.13 ± 0.66^b^	809.16 ± 21.27^b^	3.67 ± 0.03^c^
L G WW	15.75 ± 0.49^a^	90.79 ± 2.28^c^	131.55 ± 4.04^c^	13.15 ± 0.40^a^	251.24 ± 7.22^c^	43.77 ± 1.91^b^	63.07 ± 0.63^a^	666.04 ± 8.86^d^	7.17 ± 0.06^b^
L R LW	17.37 ± 0.40^a^	118.60 ± 2.73^a^	161.05 ± 4.00^b^	12.92 ± 0.53^b^	309.94 ± 7.66^a^	38.59 ± 1.44^c^	50.00 ± 0.50^c^	802.57 ± 10.84^b^	3.84 ± 0.02^c^
L R WW	15.72 ± 0.31^a^	90.29 ± 1.98^c^	132.36 ± 1.21^c^	13.28 ± 0.47^a^	251.65 ± 3.97^c^	43.42 ± 0.98^b^	63.24 ± 0.89^a^	652.72 ± 5.12^d^	6.95 ± 0.03^b^
H G LW	12.24 ± 0.42^a^	73.65 ± 0.57^c^	85.18 ± 2.17^d^	12.15 ± 0.45^c^	183.22 ± 3.19^d^	41.82 ± 1.50^bc^	46.13 ± 0.77^c^	913.00 ± 18.19^a^	3.46 ± 0.02^c^
H G WW	12.42 ± 0.18^a^	114.62 ± 0.61^a^	152.11 ± 4.14^b^	12.54 ± 0.52^c^	291.69 ± 5.28^c^	48.74 ± 1.31^a^	53.72 ± 0.37^b^	735.11 ± 9.86^c^	9.70 ± 0.36^a^
H R LW	13.79 ± 0.40^a^	74.87 ± 0.52^c^	92.29 ± 2.11^d^	13.05 ± 0.40^b^	194.00 ± 3.03^d^	43.81 ± 1.20^b^	44.28 ± 0.30^d^	912.36 ± 14.78^a^	3.72 ± 0.03^c^
H R WW	12.67 ± 0.92^a^	15.52 ± 0.64^a^	218.06 ± 4.07^a^	12.87 ± 0.49^c^	359.12 ± 5.24^a^	51.63 ± 0.97^a^	61.67 ± 0.43^a^	735.77 ± 10.08^c^	10.18 ± 0.36^a^
Significance									
VPD	*	***	***	***	NS	***	***	***	***
C	NS	***	*	**	NS	NS	NS	NS	NS
W	*	***	NS	**	NS	***	***	***	***
VPDxCxW	NS	***	NS	***	NS	**	**	**	**

Upper Epidermis thickness (UE), Palisade Thickness (PT), Spongy Thickness (ST), Lower Epidermis Thickness (LET), Lamina Thickness (LT), Intercellular Spaces (IS), Stomatal Density (SD), Stomatal Area (SA), Vein Density (VD). Mean values and standard errors are reported. NS, *; **, and ***—not significant or significant at *p* < 0.05, 0.01, and 0.001, respectively. Different letters correspond to significantly different values (*p* < 0.05)

In particular, the interaction between the factors was significant for all the measured traits, except for UET and ST. Otherwise, for cultivar alone was significant only for PT (*p* < 0.01), LET (*p* < 0.005), ST and LT (*p* < 0.05). As for the organization of leaf lamina, no significant differences were found with respect to the UET. By contrast, the thickness of lower epidermis was always enhanced under L compared with H VPD plants, except for red plants under low-watered condition (RLW), where no significant differences were observed between L and H plants.

Many differences were found in the thickness of palisade (PT) and spongy (ST) parenchyma. PT followed a different trend under L and H VPD. More specifically, in L VPD plants the thickness increased by 25–29% under LW, compared with WW, whereas in the H VPD plants, PT was reduced by 29–31% under LW. ST followed a similar trend compared with PT with thickness increases in L VPD of 32–36% under LW compared with WW and thickness reduction in H VPD plants LW compared with WW by 35–45%.

Overall, the entire leaf lamina (LT) was thicker in red plants grown at high VPD in well-watered condition (H R WW) and in red plants grown at low VPD in low-watered condition (L R LW) compared to the other conditions, with thinnest lamina recorded in green plants grown at high VPD in low-watered condition (H G LW). Intercellular spaces (IS) ranged between 38 and 51% of the entire leaf lamina with highest values in both green and red well-irrigated plants under high VPD (H G WW and H R WW) and lowest in red plants under low VPD in low-watered condition.

Concerning stomatal traits, both SD and SA varied greatly between conditions. SD was improved under L compared with H VPD plants, except for the red plants in well-watered condition, where no significant differences were found between L and H plants. Furthermore, SD was always greater under WW than LW with increases in L WW of 15–16% compared with L LW and 32% in H WW compared with H LW. SA was always enhanced in LW compared with WW plants with increases in L of 19–22% and in H of 31–32%. Moreover, SA was higher under H than in L VPD among all treatments.

As for the organization of the veins, VD was always higher in WW plants compared with LW with increases of 25–44%. The highest values were found in both green and red H WW plants and the lowest in LW plants despite the VPD condition.

### Principal component analysis and correlation analysis

**Fig. 5:**
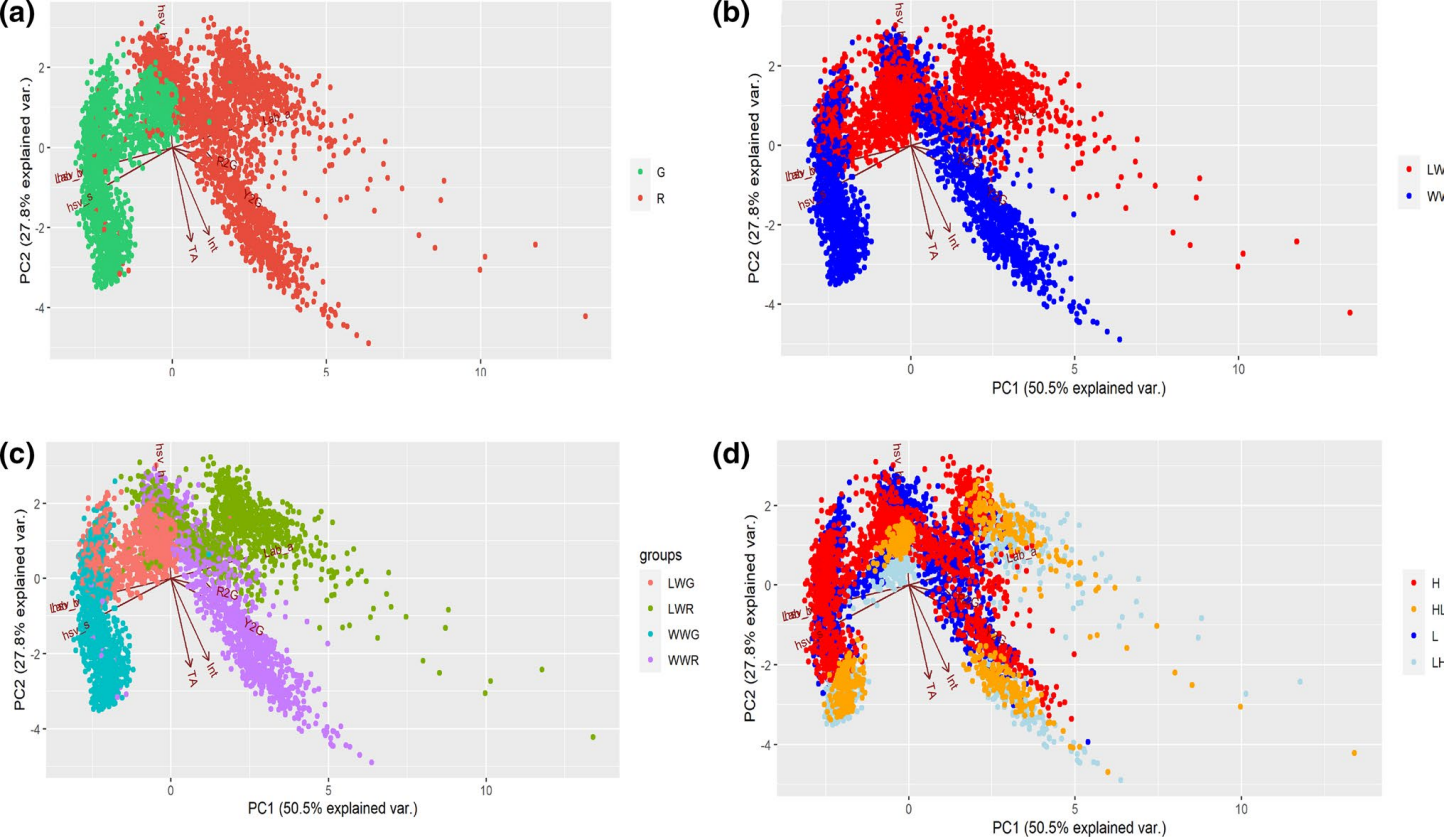
Principal component analysis (PCA) loading plot and scores of phenotypic traits in ‘Salanova’ plants separated by: (a) cultivar (Green, G; Red, R), (b) watering levels (low-watered, LW; well-watered, WW), (c) cultivar and watering levels, (d) VPD before and after changing environmental conditions (low, L; High, H; from low to high, LH; from high to low, HL)

PCA showed that for phenotypic traits the first two components cumulatively accounted for 78.3% of the total variance, with PC1 accounting for 50.5% and PC2 for 27.8%. PC1 was highly positively correlated with all variables, except for the two variables hsv_s and lab_b, while PC2 was positively correlated with hsv_s, lab_b and hsv_h (Fig. 5).

The PCA scatterplot in Fig. 5a clearly separated the cultivars (i.e., red and green ‘Salanova’) into two groups, revealing a strong clustering of green and red plants. This clustering is confirmed by the scatterplots in Fig. 5b, c, where each group is divided into sub-groups depending on the watering level (Fig. 5b) and on the watering level in combination with the cultivar (Fig. 5c), whereas no clustering was observed when the data were divided by VPD (Fig. 5d).

**Fig. 6:**
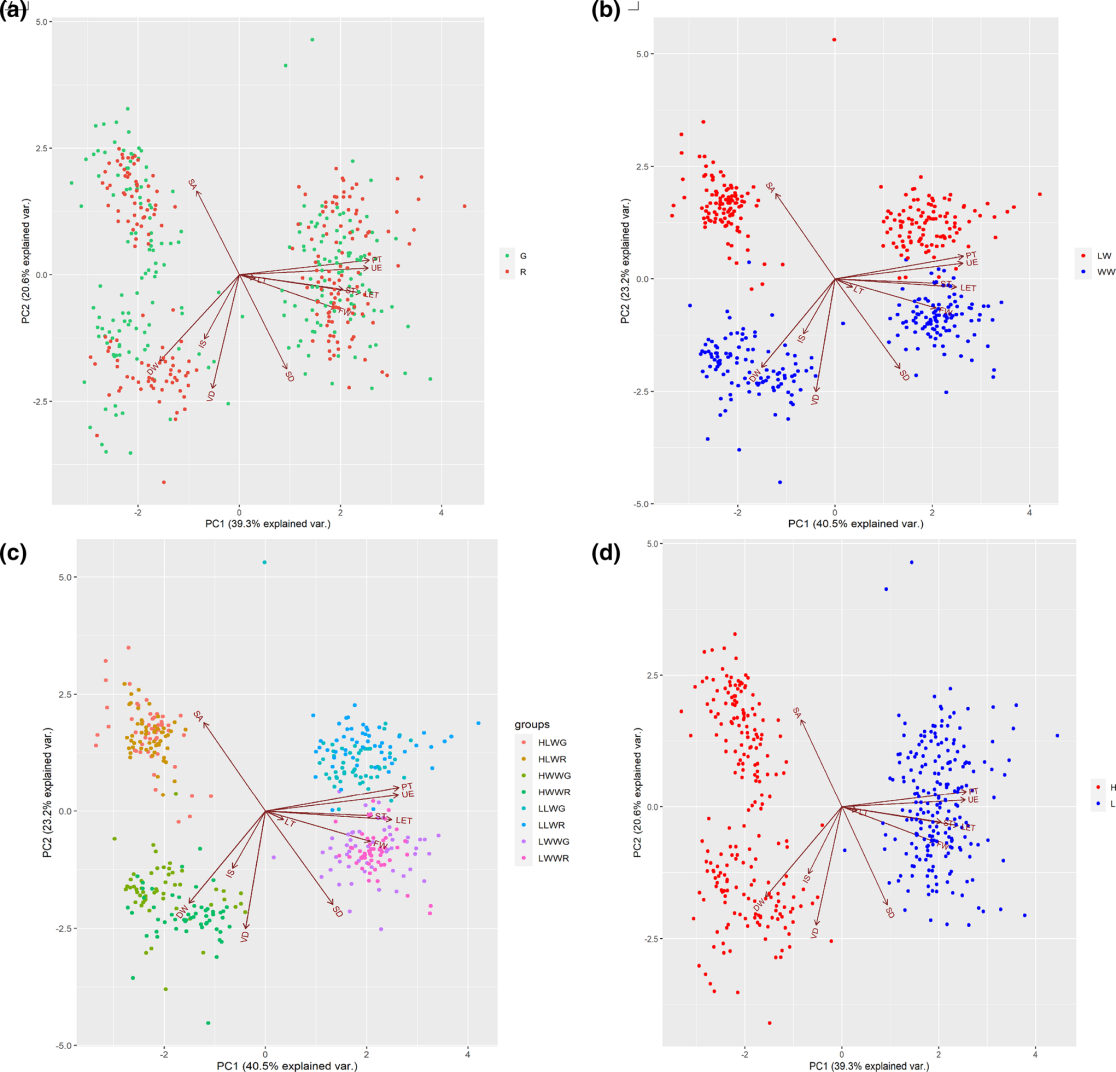
Principal component analysis (PCA) loading plot and scores of anatomical traits in ‘Salanova’ plants separated by **a** cultivar (Green, G; Red, R), **b** watering levels (low-watered, LW; well-watered, WW), **c** cultivar and watering levels, **d** VPD (low, L; High, H)

The PCA in Fig. 6 showed that for anatomical traits the first two components explained a cumulative 59.9% of the total variance with PC1 accounting for 39.3% and PC2 for 20.6%. Unlike the phenotypic traits, in Fig. 6 the anatomical traits were not separated based on the cultivar (Fig. 6a) but there was a clear distinction into clusters, when data were separated by watering levels (Fig. 6b, c) and VPD conditions (Fig. 6d).

**Fig.7:**
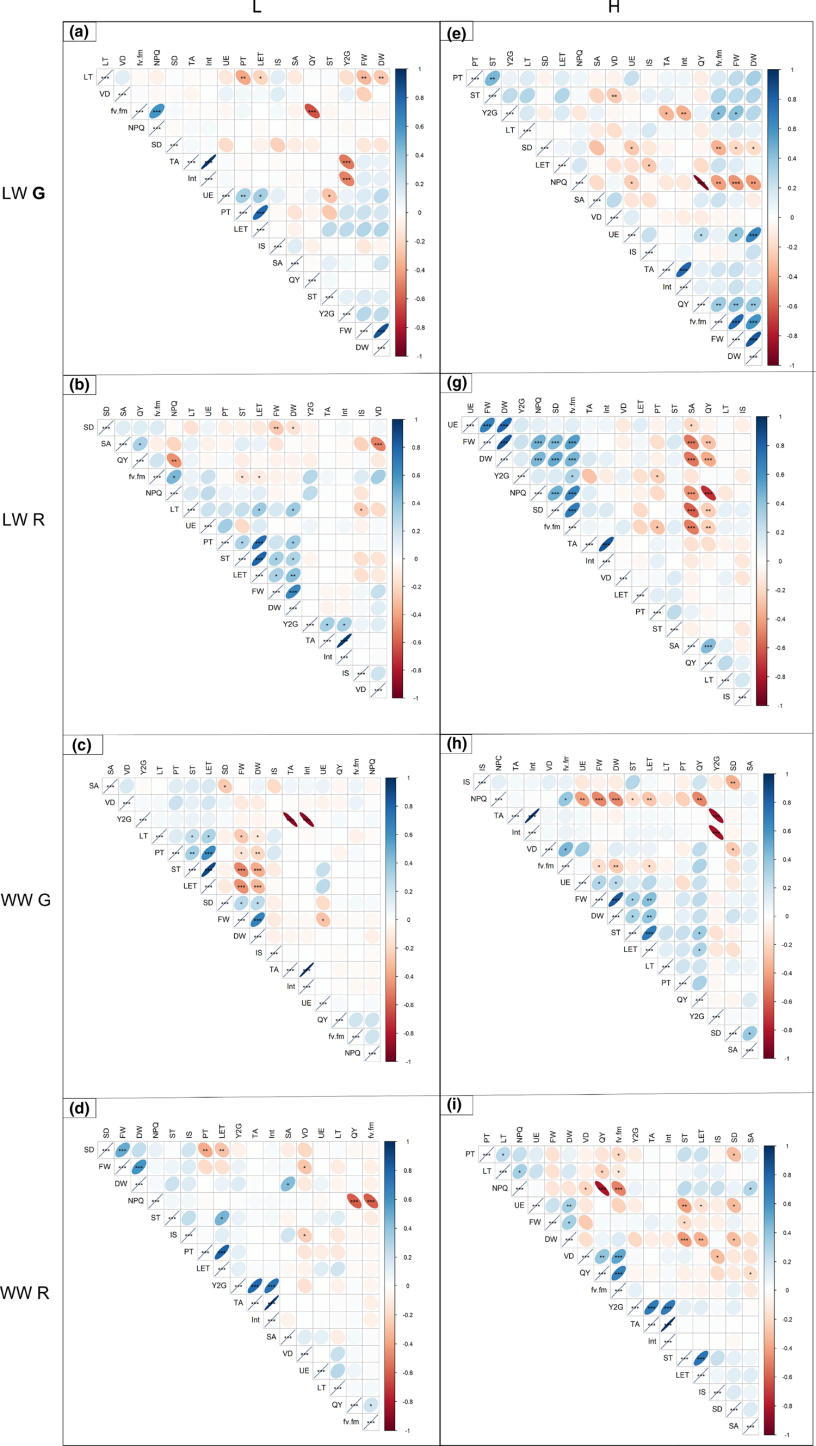
Spearman’s rank correlation coefficients between pairs of phenotypes and anatomical traits in green (G; **a**, **c**, **e**, **g**) and red (R; **b**, **d**, **f**, **h**) ‘Salanova’ plants, grown under Low (L; **a**–**d**) and High (H; **e**–**h**) VPD and irrigated with two watering levels (well-watered, WW; **c**, **d**, **g**, **h** and low-watered, LW; **a**, **b**, **e**, **f**). Positive and negative correlation are showed; *; **, and ***—significant at *p* < 0.05, 0.01, and 0.001, respectively

Many positive and negative correlations at different significant levels (*p* < 0.001 ***, *p* < 0.005**, *p* < 0.05*) were found between anatomical, biomass and phenotypic traits in plants grown at low (Fig. 7a–d) and high (Fig. 7e–h) VPD. In all treatments a strong positive correlation (*p* < 0.001, intensity level = 1) between weights (FW and DW) was always observed. Under L VPD a strong positive relationship (*p* < 0.001, intensity level = 1; Fig. 7a–d), was found between the palisade thickness (PT) and the lower epidermis thickness (LET), while the same was not observed under high VPD (Fig. 7e–h). A negative correlation was scored between NPQ and QY in all LW treatments (Fig. 7a, b, e, f), showing highest strength (*p* < 0.001) in H VPD treatments (Fig. 7e, f).

Furthermore, in G plants under both watering and VPD levels (Fig. 7a, c, e, g) it was possible to find a negative correlation between the phenotypic traits yellow to green (Y2G) and total area (TA), while in R plants (Fig. 7b, d, f, h) this correlation was positive.

### Phenotyping traits and PSII machinery after environmental changes

Table 3 reports the phenotyping results of imaging fluorimeters showing PSII performance before and after the change in environmental conditions. As main factors, VPD (V), cultivar (C) and water level (W) always had a significant effect, also showing a significant effect in combination (VPD × C × W) with a *p* value that was always < 0.01. *F*_v_/*F*_m_ values have always been higher under L VPD despite cultivar (G and R) and water regimens (WW and LW). Overall highest values were found in L WW plants compared with L LW (reduction of 1.2–3.6% compared with WW). LH plants showed reduced Fv/Fm values compared with L (reduction of 2.4%), but in any case, higher or at least comparable with H and HL. H VPD plants showed lowest *F*_v_/*F*_m_ values, especially under LW (reduction of 2.5–3.7% compared with WW). HL plants showed higher values than H plants (approximately 2% increase). As for the differences in cultivars, compared with L WW, G WW plants showed higher values under LH and lower H and HL, whereas no differences were found between green and red plants grown under low VPD and low-watered condition (LGWW and LRWW). In contrast, G LW plants showed higher values under LH and lower under L and HL; no differences were detected in H compared with R LW. The Φ_PSII_ values were highest under L VPD in all conditions, except for G LW, where no differences were found between L and LH plants. The overall highest values were found in L WW plants compared with L LW (reduction of 16% compared with WW). LH plants showed reduced values compared with L, except under G LW, but in any case, higher or at least comparable with H and HL. H VPD plants showed reduced Φ_PSII_ values compared with LVPD, especially under LW (20% reduction compared with L G LW). HL plants showed higher values compared with H, except for GWW, where no statistically significant differences were observed between H, LH and HL. NPQ values were always enhanced under LW condition, in HL, H, LH and L plants. As for the red cultivar, the plants always showed higher NPQ values than the green plants. Table 4 reports the phenotyping results of selected traits in VIS, IR and FLUO imaging fluorimeters showing PSII performance before and after changing environmental conditions. As main factors, VPD (V), cultivar (C) and water level (W) always had a significant effect, except for hsv_s, hsv_v and hsv_h were VPD alone was not significant. Moreover, their interaction (VPD × C × W) also showed a significant effect on all parameters, except for hsv_s, hsv_v and hsv_h and R2G, with a *p* value < 0.001 in Y2G and Int and < 0.05 in lab_a and lab_b. More specifically, Y2G, lab_b and Int exhibited higher values in green plants grown at high VPD in low-watered conditions. Hsv_s and hsv_v exhibited higher values in green plants at well-watered conditions in both L and H VPDs before and after the change in conditions. As for the R2G trait, higher values were found in red plants under low watered after changing the environment from high to low VPD.

**Table 3:** PSII performance of green (G) and red (R) ‘Salanova’ plants grown at the two watering levels (well-watered, WW; low-watered, LW) under Low (L) and High (H) VPD before and after the change in environmental conditions (from low to high, LH; from high to low, HL)

	*F*_v_/*F*_m_	Φ_PSII_	NPQ
L			
L G LW	0.85 ± 0.002^c^	0.51 ± 0.003^b^	0.97 ± 0.20^m^
L G WW	0.87 ± 0.002^a^	0.63 ± 0.008^a^	0.44 ± 0.01^e^
L R LW	0.85 ± 0.005^b^	0.49 ± 0.005^b^	1.18 ± 0.02^cd^
L R WW	0.87 ± 0.001^a^	0.55 ± 0.007^a^	0.64 ± 0.05^i^
LH			
LH G LW	0.84 ± 0.001^d^	0.51 ± 0.006^b^	1.12 ± 0.07^d^
LH G WW	0.85 ± 0.006^c^	0.57 ± 0.003^ab^	0.73 ± 0.02^h^
LH R LW	0.83 ± 0.008^e^	0.47 ± 0.004^c^	1.23 ± 0.03^c^
LH R WW	0.84 ± 0.007^d^	0.51 ± 0.003^b^	0.84 ± 0.01^g^
H			
H G LW	0.81 ± 0.006^f^	0.46 ± 0.009^e^	1.16 ± 0.04^d^
H G WW	0.84 ± 0.003^d^	0.59 ± 0.003^b^	0.53 ± 0.01^i^
H R LW	0.82 ± 0.009^f^	0.39 ± 0.006^f^	1.44 ± 0.03^ab^
H R WW	0.84 ± 0.009^d^	0.50 ± 0.003^c^	0.84 ± 0.02^g^
HL			
HL G LW	0.82 ± 0.008^f^	0.47 ± 0.009^d^	1.31 ± 0.06^b^
HL G WW	0.84 ± 0.005^d^	0.59 ± 0.003^b^	0.59 ± 0.01^i^
HL R LW	0.83 ± 0.006^e^	0.43 ± 0.009^d^	1.53 ± 0.05^a^
HL R WW	0.84 ± 0.006^d^	0.52 ± 0.002^c^	0.88 ± 0.02^f^
Significance			
VPD	***	***	***
C	***	**	**
W	***	***	***
VPD × C × W	***	**	**

Maximum photochemical efficiency (*F*_v_/*F*_m_), the quantum yield of PSII electron transport (Φ_PSII_), non-photochemical quenching (NPQ). Mean values and standard errors are reported. NS, *; **, and ***—not significant or significant at *p* < 0.05, 0.01, and 0.001, respectively. Different letters correspond to significantly different values (*p* < 0.05)

**Table 4:** Selected phenotipic traits in terms of Yellow to Green (Y2G), Lab color a (Lab_a), Lab color b (Lab_b) and intensity mean (Int) of green (G) and red (R) ‘Salanova’ plants grown under two different water regimens (WW and WL) and under low (L) and high (H) VPD before (L, H) and after (LH, HL) changes in environmental conditions. Mean values and standard errors are reported

	Y2G	R2G	lab_a	lab_b	hsv_s	hsv_v	hsv_h	Int
L G WW	0.00019^f^	0.000013^b^	98.32^g^	142.68^d^	0.70^a^	0.46^a^	0.25^d^	0.63^i^
L G LW	0.00014^f^	0.0000294^b^	98.36^g^	133.41^g^	0.61^b^	0.33^b^	0.27^b^	0.69^f^
L R WW	0.00107^f^	0.0042^b^	106.889^e^	143.73^d^	0.57^d^	0.20^d^	0.24^e^	0.63^i^
L R LW	0.00007^f^	0.0107^b^	105.28^f^	138.44^f^	0.45^f^	0.17^ef^	0.28^b^	0.72^d^
H G WW	0.00738^f^	0.0000989^b^	115.75^d^	150.91^c^	0.71^a^	0.45^a^	0.25^d^	0.70^e^
H G LW	0.3520^a^	0.0000301^b^	116.35^cd^	166.75^a^	0.57^b^	0.29^b^	0.28^b^	0.83^a^
H R WW	0.16603^b^	0.012058^b^	116.82^cd^	139.96^e^	0.54^d^	0.16^d^	0.23^e^	0.74^c^
H R LW	0.15656^bc^	0.050426^a^	117.40b^c^	166.44^a^	0.32^f^	0.14^ef^	0.27^a^	0.82^b^
LH G WW	0.00004^f^	0.0000237	97.94^g^	165.63^a^	0.71^a^	0.46^a^	0.25^d^	0.66^h^
LH G LW	0.03^f^	0.0000323^b^	107.47^e^	152.86^b^	0.58^c^	0.32^c^	0.28^b^	0.72^cd^
LH R WW	0.014352^d^	0.00210^b^	118.26^b^	138.24^f^	0.56^e^	0.19^g^	0.24^f^	0.68^g^
LH R LW	0.19924^ab^	0.03483^a^	122.11^a^	149.39^c^	0.44^g^	0.18^f^	0.29^c^	0.73^cd^
HL G WW	0.00008^f^	0.0000107^b^	98.02^g^	165.27^a^	0.71^a^	0.45^a^	0.25^d^	0.62^i^
HL G LW	0.00061^f^	0.0000313^b^	107.67^e^	149.51^c^	0.57^c^	0.29^c^	0.28^b^	0.72^cd^
HL R WW	0.13095^cd^	0.00699^b^	117.47^bc^	140.30^e^	0.55^e^	0.17^f^	0.24^e^	0.65^h^
HL R LW	0.12650^cd^	0.04035^a^	121.01^a^	134.72^g^	0.35^g^	0.15^f^	0.26^c^	0.74^c^
Significance								
VPD	**	*	*	**	NS	NS	NS	*
C	***	***	***	**	**	**	**	***
W	***	**	***	***	*	**	**	***
VPD × C × W	***	NS	*	*	NS	NS	NS	***

NS, *; **, and ***—not significant or significant at *p* < 0.05, 0.01, and 0.001, respectively. Different letters correspond to significantly different values (*p* < 0.05)

## Discussion

### *The combination of VPD and water levels influences the development and the morpho-anatomical traits of the leaves*.

Changes in VPD and water levels during plant growth strongly influenced biomass allocation and the morpho-anatomical organization of the leaves in green and red ‘Salanova’ plants. In previous high-throughput phenotyping studies (Yang et al. 2009; Dodig et al. 2019), RGB imaging, including plant area and convex hull area, were used to predict plant biomass in many crops (especially corn, barley, and wheat). Here, biomass data in terms of fresh and dry weight measured manually in all plants at the end of the experiment (Table 1) were in good agreement with the plant area estimated by RGB cameras (Fig. 3), showing the highest area and weight (both fresh and dry) in well-watered plants under low VPD (L WW) and the lowest in low-watered plants under high VPD (H LW). Indeed, it is known that the reduction of VPD in the air by humidification significantly increases the biomass allocation in the leaves and in the fruits (Zhang et al. 2017). Furthermore, a larger leaf area positively affects the transpiration rate during growth, also favoring a balanced absorption of nutrients and a correct water supply to the plant (Pons et al. 2001). Interestingly, in the present study after the switch in environmental conditions, only the red cultivar under high VPD (HL R WW) managed to reach values of plant area similar to the red cultivar at low VPD, in well-watered conditions (Fig. 3b, c). The short-time exposure to low VPD probably boosted their development in terms of area expansion, but it was not yet sufficient to improve the fresh and dry weight, which in high VPD plants was still lower than the low VPD condition.

Increases in plant area and in biomass in the red ‘Salanova’ cultivar compared with the green one has been reported in earlier studies and explained as the red ‘Salanova’ reached maturity earlier than the green one (Amitrano et al. 2021b; El-Nakhel et al. 2019).

In the present study, the red cultivar under well-watered conditions also showed a higher stomatal density than the green one (Table 2). Indeed, a positive correlation (*p* < 0.001) was observed between the stomatal density (SD) and the fresh weight (FW) (Fig. 7d). This probably explains the improvements in leaf area when red plants developed at high VPD, where subjected to short-term low-VPD (more favorable environmental conditions). Higher stomatal density is known to improve carbon gain and maintain carbon gain/water loss homeostasis in plants (Lawson and Blatt 2014). However, in WWR plants between SD and FW there was a tendency toward positive correlation that, however, was not statistically significant (Fig. 7h).

Otherwise, the lack of fresh and dry weight gain in well-watered red plants at high VPD (H R WW) (together with the area) could probably be ascribed to the greater development of spongy tissue in these plants, compared with other treatments. As also confirmed by the negative correlation (*p* < 0.05) between SD and DW (Fig. 7h). The spongy parenchyma, in fact, is richer in intercellular spaces with cells that develop slightly thinner cell walls, compared with the palisade parenchyma (Fan et al. 2013). In these plants it was also possible to score a negative correlation between the spongy thickness (ST) and the DW (Fig. 7h).

Overall, in the present study the combination of elevated VPD and water stress induced the development of an anatomical leaf structure in both green and red plants that probably increased the resistance of the mesophyll to water flow and was responsible for a reduced ability in acclimation of these plants when subjected to short-term changes in environmental conditions. For example, a higher percentage of intercellular spaces is a clear sign of greater resistance to water flows in leaves, due to the reduced connection of the cells and the greater presence of airspaces, which increased the length of the path, also reducing the speed of the flows (Amitrano et al. 2019).

Moreover, the lower stomatal density and larger stomatal area could be one of the main reasons of the lower yield of these plants and of the reduced acclimation capacity when subjected to changing conditions, since they did not allow the plants to efficiently regulate the transport and transpiration of water (Dickison 2000). Indeed, the proportion of anatomical traits and in particular of the palisade and spongy parenchyma and of the stomatal and vein density can vary in relation to plant species and habitat, being strictly influenced by the surrounding environment and can affect plant photosynthesis and the entire eco-physiological behavior (Esau 1965). Here, together with a larger size of the fewer stomata and a higher percentage of intercellular spaces, a lower vein density was also observed in all plants subjected to water stress, irrespective of the VPD condition. The veining of the leaves is essential for water supply; an extended vascular system manages to maintain water balance even when transpiration is high (Brodribb and McAdam 2017; Liu et al. 2016). Different authors proposed that in species with high leaf plasticity, which promptly adapt to changes in environmental conditions, stomatal and vein densities should be coordinated to allow the maintenance of a high physiological efficiency (photosynthesis, conductance, water use efficiency) of the species under sub-optimal environmental conditions (Brodribb et al. 2007; Carins Murphy et al. 2012). However, contrasting results were found for leaf size, vein, and stomatal density under high and low VPDs. Here, at high VPD there was a different response between well- and low-watered plants: while in well-watered plants, the stomatal and vein densities increased, probably to try to compensate for the air drought, in the low-watered plants the reduced stomatal density together with the reduced vein density might be the cause behind the lower biomass production and the reduced capacity in acclimation of these plants when subjected to the changing VPD.

### Photosystem acclimation to changing VPD is guided by the anatomical traits of the leaves

Since high-throughput measurements of photosynthesis are challenging, a way to quickly estimate the health of the photosynthetic apparatus when subjected to environmental stress is through chlorophyll fluorescence quenching parameters, such as electron transport rate, photodamage, or the intrinsic photochemical efficiency of light harvesting in photosystem II (*F*_v_/*F*_m_) (Baker 2008; Baker and Rosenqvist 2004).

In the present study it was possible to observe statistically significant differences in the PSII performance (*F*_v_/*F*_m_, Φ_PSII_, NPQ) between well- and low-watered plants grown at low and high VPD (Table 3). Differently, in previous studies using measurement of chlorophyll “*a*” fluorescence, *F*_v_/*F*_m_ was relatively insensitive to drought stress. Just to mention a few, Munns et al. (2010) found no differences in the *F*_v_/*F*_m_ time course of both well-irrigated and drought-prone barley plants.

In rice, the *F*_v_/*F*_m_ parameter did not significantly change in the early response to drought stress (Kim et al. 2020). Similarly, in our study the *F*_v_/*F*_m_ values were always above 0.8 (even in LW plants), which is considered a threshold for stressed plants (Ogaya et al. 2011). This may be because plants in the first phase of development showed resistance to water stress, implementing morphological adjustments that led to the reduction of their yield and biomass but allowed them to survive without permanent damage (Yang et al. 2021).

After the switch in environmental conditions, both F_v_/F_m_ and Φ_PSII_ showed the same trend, with reduction moving from low to high VPD (L to HL) and enhancement in values from high to low VPD (H to LH); however, this trend was more evident in *F*_v_/*F*_m,_ where the differences were always statistically significant (Table 3). Conversely, the NPQ, an indicator of the light energy dissipated in heat to preserve the integrity of photosystem II (Ruban 2016), followed an opposite trend, being higher under LW and high VPD (H and LH).

We attributed these differences in the photosynthetic apparatus to the different organization of the leaf tissue. Indeed, the reduction of stomatal density with increases in water deficit are common in several species (Orsini et al. 2011; Waqas et al. 2017) and together with a reduced opening of the pores are the most common causes of reduction of the photosynthesis and conductance under drought stresses (Domec et al. 2017; Kelly et al. 2016). By contrast, leaf vein traits are even less studied and their development under drought stress has not been investigated much, so far. In addition, in our study, low-VPD plants, especially under limiting water, developed a thicker palisade mesophyll. This trait together with a high stomatal density (compared with H plants) was probably the reason for the maintaining a greater photochemical efficiency even after the switch in environmental conditions (LH). In fact, looking at the related physiological traits, LH plants maintained *F*_v_/*F*_m_ and Φ_PSII_ values higher or at least comparable with H and LH plants.

However, to the best of our knowledge, this different VPD-driven acclimation of lettuces under water stress has never been reported before and is likely to have a major influence on photosynthesis and plant acclimation to changing environmental conditions that cannot be easily detected with RGB cameras.

### The limits of phenotyping in detecting the anatomical differences of plants

Non-destructive assessment of plants can provide valuable information on their growth and acclimation to environmental conditions. In this study, phenotypic traits revealed early stress in LW plants, especially under high VPD. For example, the Y2G (yellow to green) increased in low-watered plants under H and LH VPD compared with well-watered plants (Table 4), remarking a stressful condition.

Yellow to green (Y2G) is one of the color-related traits most sensitive to drought stress, which can also reveal symptoms of wilting and senescence (Neumann et al. 2015). In agreement with Y2G, the same plants showed improved Lab_b. High value of this trait indicates the yellow color and Lab_b is, therefore, a proxy for the stress level. Furthermore, several studies (Dodig et al. 2019; Ibraheem et al. 2012) have found a relationship between increase in Lab_b and decrease in dry weight in different cultivars.

The same relationship is reported in this study, particularly for H plants. The main response of plants to drought and high evaporative demand was undoubtedly a reduction in plant area, which likely translates into a decrease in water content of plants. Furthermore, a previous study on rice found a strong correlation between plant area and NIR intensity (indicator of plant water content) (Kim et al. 2020). In agreement with this study, here the Int trait (NIR intensity) was always positively correlated with plant total area (TA) (Fig. 7) and it resulted lower in HVPD and LW plants, indicating lower water content in ‘Salanova’ lettuces, compared with the other treatments (Table 4).

Also, as expected, the Lab_a trait was improved in R plants as a high value of this trait indicates the red color and small values indicate the green color. Lab_a was also boosted under LW, indicating increased redness of water-stressed plants.

However, as highlighted by the multivariate analysis in Fig. 5 on phenotypic (RGB, NIR, FLUO) traits, the samples grouped by color (Fig. 5a) and by water availability (Fig. 5b), while the different VPD seems to have no effect on the grouping of data in the PCA (Fig. 5d).

Otherwise, by performing a second multivariate analysis on the anatomical traits, it was evident how the samples were clearly grouped by VPD (Fig. 6d) and by water availability (Fig. 6b) and that the influence of color was minimal (Fig. 6a). Thus, bearing in mind that in the present study fluorescence imaging of the chlorophyll “*a*” revealed many differences between plants grown under different VPDs both before and after the change of conditions, we can, therefore, conclude that these physiological differences in PSII performance were due to anatomical differences between treatments, which cameras (with the exception of chlorophyll “*a*” fluorescence) were not actually able to detect. Indeed, these cameras could easily detect the stress already in progress in plants but were not able to detect differences in their structural organization, which were responsible for the greater or fewer capacity in acclimation in case of sudden environmental fluctuation.

Therefore, these combined analyses (phenotyping and tissue anatomy) could serve as a proxy for early diagnosis of environmental stress even before symptoms occur (Chen et al. 2012; Humplík et al. 2015; Janka et al. 2018; Tschiersch et al. 2017) and fill the still existing knowledge gap in functional phenomics concerning the relationships between plant phenotype and functional anatomical traits (Kim et al. 2020) which could, therefore, be used as key indicators of photosynthetic efficiency (Baker and Rosenqvist 2004).

## Conclusions

In the current context of climate change, very rapid shifts in environmental conditions are expected; therefore, the understanding of the morpho-physiological acclimation mechanisms of crops is necessary to acquire knowledge to improve the cultivation techniques both in the open-field and in controlled-environment agriculture, where sudden climate fluctuation are due to environmental control systems.

In this context, many high-throughput phenotyping experiments have investigated plant responses to various environmental stresses, such as drought, salinity, extreme heat, and frost events (Campbell et al. 2015; Hairmansis et al. 2014). However, to the best of our knowledge, none have used high-throughput phenotyping in combination with microscopy analysis of plant tissue anatomy to study the combined effect of VPD (low and high) and of two different water levels (well-watered and low-watered) especially with the aim of detecting early stress signals in lettuce plants during the early stages of development, focusing on the morpho-physiological acclimation of the plant to the change in VPD.

The main findings of this study showed that different VPDs and watering levels determine a different anatomical leaf structure in lettuce, which influenced the extent of crop acclimation. Phenotyping imaging techniques, although effective and reliable, should be complemented with structural or molecular measurements to get the complete picture of crops’ plasticity, which is driven by crop anatomical development and influenced by environmental conditions. Indeed, while a combination of RGB, fluorescence and thermal imaging can provide a powerful tool for the early diagnosis of stress symptoms (including air drought and water stress), it is, therefore, essential to support high-throughput phenotyping with the quantification of leaf anatomical traits at specific stages of plant development to forecast the anatomical-mediated ranges for plant physiological acclimation, finally modelling the transient adaptation of the plant to a changing microclimate.

### *Author contribution statement*

Conceptualization (VDM, CA, AJ), Methodology (AJ, VDM, CA, NDA), data curation (VDM, NDA, CA), original draft preparation (CA), visualization (CA, VDM, NDA), investigation (CA), supervision (VDM, AJ, SDP), writing—reviewing and editing (CA, VDM, AJ, NDA, SDP)**.**

## Supplementary Information

Below is the link to the electronic supplementary material.

Supplementary file1 (DOCX 116 KB)

## Data Availability

The data supporting the findings of this study are available from the corresponding author, upon reasonable request.
