# Supplementary material for: Integration of high-throughput phenotyping with anatomical traits of leaves to help understanding lettuce acclimation to a changing environment
Source: Planta. 2022 Sep 2;256(4):68. doi: 10.1007/s00425-022-03984-2 (PMC9439985; doi:10.1007/s00425-022-03984-2)
Supplement: Supplementary file 1 — Supplementary file1 (DOCX 116 KB) [file 425_2022_3984_MOESM1_ESM.docx]

**Supplemental materials**

**Table S1.**List of selected traits. Name, description and category are specified.

| **Trait Name** | **Trait Description** | **Trait code** | **Trait category** |
| --- | --- | --- | --- |
| Area | Projected plant pixel area from top view. It may serve as a measure of plant size and correlates with plant biomass. | TA | Architectural |
| Yellow to green | Proportion of yellow color plant pixels divided by the count of green color pixels. Measure of amount of senescent tissues (yellow). | Y2G | Color-related |
| Lab color a | Average color in the a* range (green to red) of the L* a* b* color space. Small values indicate green and high values indicate red. Indicator of level of stress. | Lab_a | Color-related |
| Lab color b | Average color in the b* range (blue to yellow) of the L* a* b* color space. Small values indicate blue and high values indicate yellow. High values are Indicator of level of stress. | Lab_b | Color-related |
| Red to green | Proportion of red color plant pixels (histogram bins 0 and 1) from side view divided by the count of green color pixels (bins 4 to 7) from side view. Indicator of level of stress. | R2G | Color-related |
| Mean fluorescence | Color-related trait based on fluorescence view. Mean - first order texture property (independent of pixel neighbors). Calculated on grayscale image derived from channel Brightness (HSV). | hsv_s | Color-related |
| Mean hue | Color-related trait based on visible light view. Mean - first order texture property (independent of pixel neighbors). Calculated on grayscale image derived from channel Hue (HSV). | hsv_h | Color-related |
| Mean brightness | Color-related trait based on visible light view. Mean - first order texture property (independent of pixel neighbors). Calculated on grayscale image derived from channel Brightness (HSV). | hsv_v | Color-related |
| Intensity mean | Average intensity of the fluorescence reflection based on the color of each pixel (pure red highest intensity, yellow lowest intensity). Provides a relative measure of photosynthetic health by detecting senescence, necrosis and chlorosis. | Int | Physiological and Color-related |
| Quantum yield of Photosystem II (PSII) | The operating efficiency of PSII photochemical, F_q_′/F_m_′, calculated from (F_m_′–F′)/F_m_′.  It gives the proportion of absorbed light that is used in PSII photochemical and can be used to estimate the rate of electron transport through PSII with knowledge of light absorptance by the leaf and photosystems. | Φ_PSII_ | Physiological |
| Maximum efficiency of Photosystem II (PSII) | Ratio of variable to maximum fluorescence—the quantum efficiency of open photosystem II centers. | F_v_/F_m_ | Physiological |
| Non-photochemical quenching | Non-photochemical processes that dissipate excitation energy. | NPQ | Physiological |

**Table S2**. Spearman’s correlations between pairs of phenotypes of green (G) ‘Salanova’ plants grown under low-watered (LW) regimes and low (L) VPD. P-values in the upper-right corner, rho values in the bottom-left corner.

| **TA** | **Y2G** | **Int** | **FW** | **DW** | **UE** | **PT** | **ST** | **LET** | **LT** | **IS** | **SD** | **SA** | **VD** | **QY** | **fv.fm** | **NPQ** |  |
| --- | --- | --- | --- | --- | --- | --- | --- | --- | --- | --- | --- | --- | --- | --- | --- | --- | --- |
| **TA** | 0 | 4.76E-05 | 3.35E-19 | 0.883974 | 0.979754 | 0.766895 | 0.5589 | 0.867272 | 0.437367 | 0.997151 | 0.651719 | 0.90318 | 0.691581 | 0.426885 | 0.983512 | 0.767241 | 0.975457 |
| **Y2G** | -0.52297 |  | 5.92E-05 | 0.14492 | 0.132415 | 0.487391 | 0.132141 | 0.651943 | 0.069849 | 0.142892 | 0.727671 | 0.804599 | 0.795057 | 0.838506 | 0.921694 | 0.954966 | 0.603376 |
| **Int** | 0.964995 | -0.49029 | 0 | 0.88513 | 0.992029 | 0.790669 | 0.560743 | 0.850594 | 0.438735 | 0.976618 | 0.724655 | 0.976332 | 0.646911 | 0.520543 | 0.976369 | 0.754985 | 0.949889 |
| **FW** | 0.107982 | 0.270151 | 0.12337 | 0 | 4.53E-10 | 0.274923 | 0.126703 | 0.499956 | 0.054815 | 0.004457 | 0.197705 | 0.293772 | 0.835984 | 0.065417 | 0.928184 | 0.641096 | 0.682305 |
| **DW** | 0.095238 | 0.259332 | 0.115372 | 0.864875 |  | 0.157435 | 0.16452 | 0.617261 | 0.078694 | 0.006812 | 0.323127 | 0.172474 | 0.437256 | 0.25713 | 0.887544 | 0.519883 | 0.519785 |
| **UE** | 0.024014 | 0.084043 | 0.031504 | 0.14052 | 0.263087 | 0 | 0.00982 | 0.030591 | 0.032341 | 0.142998 | 0.718207 | 0.10535 | 0.622522 | 0.859106 | 0.671468 | 0.605252 | 0.664064 |
| **PT** | -0.02062 | 0.209478 | -0.01787 | 0.216817 | 0.186078 | 0.333766 |  | 0.177623 | 9.07E-07 | 0.004937 | 0.543051 | 0.694976 | 0.287351 | 0.886959 | 0.716579 | 0.898081 | 0.774972 |
| **ST** | 0.025068 | 0.1088 | 0.026902 | 0.154539 | 0.131593 | -0.28915 | -0.26597 | 0 | 0.826149 | 0.904889 | 0.625358 | 0.980474 | 0.451326 | 0.519495 | 0.813454 | 0.560953 | 0.52722 |
| **LET** | -0.0082 | 0.269286 | 0.005454 | 0.292609 | 0.290201 | 0.348504 | 0.793084 | 0.252316 |  | 0.013425 | 0.494761 | 0.435399 | 0.417636 | 0.719045 | 0.894104 | 0.758497 | 0.445069 |
| **LT** | -0.02882 | -0.17544 | -0.01407 | -0.32809 | -0.26558 | -0.12162 | -0.38722 | 0.022365 | -0.21146 | 0 | 0.314633 | 0.723733 | 0.596015 | 0.303943 | 0.915645 | 0.870984 | 0.726826 |
| **IS** | -0.02923 | -0.00588 | 0.012978 | -0.12237 | -0.05198 | 0.118203 | -0.02896 | -0.01272 | -0.00339 | 0.12568 | 0 | 0.083337 | 0.437408 | 0.3245 | 0.620385 | 0.859071 | 0.820209 |
| **SD** | 0.041801 | 0.029498 | 0.022989 | -0.12753 | -0.13609 | -0.18087 | 0.00559 | 0.015136 | -0.06081 | 0.015914 | -0.2317 | 0 | 0.361696 | 0.765961 | 0.543344 | 0.767091 | 0.864337 |
| **SA** | -0.01395 | 0.047006 | -0.03541 | 0.016763 | 0.207522 | -0.02754 | -0.14156 | 0.116181 | -0.06315 | -0.11441 | 0.139845 | -0.09766 | 0 | 0.961815 | 0.960925 | 0.569323 | 0.591644 |
| **VD** | -0.05539 | 0.09282 | 0.009104 | -0.23172 | 0.001719 | 0.085684 | 0.050105 | -0.0248 | 0.048577 | 0.128768 | 0.138142 | -0.02611 | 0.032328 | 0 | 0.842577 | 0.973681 | 0.533188 |
| **QY** | 0.002525 | -.0003 | 0.013221 | 0.007363 | 0.025617 | 0.086477 | -0.05348 | 0.040104 | -0.00178 | 0.010706 | -0.07134 | -0.0878 | -0.01045 | -0.00836 | 0 | 4.53E-05 | 0.238574 |
| **fv.fm** | -0.0242 | 0.021669 | -0.01912 | -0.03211 | -0.04016 | -0.04512 | 0.053551 | -0.04961 | 0.007334 | -0.02928 | 0.042858 | 0.034403 | -0.06812 | 0.029103 | -0.63246 | 0 | 0.000357 |
| **NPQ** | 0.036793 | -0.03999 | 0.040958 | 0.01353 | 0.00782 | 0.014745 | 0.000601 | -0.02701 | -0.02079 | -0.0711 | 0.002244 | -0.02803 | -0.02191 | -0.03941 | 0.00098 | 0.6 | 0 |

**Table S3**. Spearman’s correlations between pairs of phenotypes of red (R) ‘Salanova’ plants grown under low-watered (LW) regimes and low (L) VPD. P-values in the upper-right corner, rho values in the bottom-left corner.

| **TA** | **Y2G** | **Int** | **FW** | **DW** | **UE** | **PT** | **ST** | **LET** | **LT** | **IS** | **SD** | **SA** | **VD** | **QY** | **fv.fm** | **NPQ** |  |
| --- | --- | --- | --- | --- | --- | --- | --- | --- | --- | --- | --- | --- | --- | --- | --- | --- | --- |
| **TA** | 0 | 0.036066 | 2.16E-17 | 0.231434 | 0.153947 | 0.674881 | 0.263642 | 0.390015 | 0.274014 | 0.359326 | 0.978598 | 0.971895 | 0.802629 | 0.90014 | 0.754973 | 0.817552 | 0.854127 |
| **Y2G** | 0.336987 |  | 0.038835 | 0.362032 | 0.206855 | 0.924187 | 0.232937 | 0.192911 | 0.167568 | 0.35924 | 0.660046 | 0.632307 | 0.281466 | 0.413038 | 0.248497 | 0.136889 | 0.136656 |
| **Int** | 0.950913 | 0.335364 | 0 | 0.237248 | 0.170344 | 0.749293 | 0.266064 | 0.370964 | 0.267579 | 0.374818 | 0.983773 | 0.946497 | 0.769346 | 0.932625 | 0.695701 | 0.762338 | 0.842961 |
| **FW** | -0.04765 | 0.01283 | -0.05761 | 0 | 1.47E-06 | 0.494255 | 0.093977 | 0.030631 | 0.033465 | 0.216502 | 0.95188 | 0.00707 | 0.292718 | 0.395671 | 0.273585 | 0.107281 | 0.652063 |
| **DW** | -0.06574 | -0.00761 | -0.04122 | 0.651835 |  | 0.561004 | 0.020536 | 0.014051 | 0.00638 | 0.037637 | 0.621012 | 0.017492 | 0.641821 | 0.858015 | 0.34356 | 0.057723 | 0.671889 |
| **UE** | 0.017387 | 0.065966 | 0.050604 | -0.00394 | -0.01838 | 0 | 0.211631 | 0.254291 | 0.905651 | 0.408398 | 0.488534 | 0.927731 | 0.38292 | 0.916226 | 0.484547 | 0.315641 | 0.092863 |
| **PT** | 0.005975 | 0.009692 | -0.00348 | 0.203401 | 0.366888 | 0.341398 |  | 0.012188 | 6.38E-06 | 0.089283 | 0.304725 | 0.111601 | 0.747282 | 0.584913 | 0.529182 | 0.118111 | 0.810028 |
| **ST** | -0.00204 | -0.05105 | -0.01377 | 0.330596 | 0.307752 | -0.19484 | 0.304862 | 0 | 5.06E-07 | 0.055232 | 0.07479 | 0.133243 | 0.467176 | 0.148196 | 0.395966 | 0.017111 | 0.993972 |
| **LET** | 0.007922 | -0.01116 | -0.01246 | 0.327262 | 0.415726 | 0.141956 | 0.774476 | 0.805581 |  | 0.016458 | 0.063064 | 0.071097 | 0.685714 | 0.17763 | 0.405937 | 0.023735 | 0.821252 |
| **LT** | -0.00231 | 0.008046 | 0.007954 | 0.173493 | 0.365164 | 0.212337 | 0.205656 | 0.236379 | 0.364891 | 0 | 0.011878 | 0.077359 | 0.454908 | 0.078184 | 0.844451 | 0.3745 | 0.471189 |
| **IS** | 0.024705 | 0.07447 | 0.00152 | 0.0544 | 0.033154 | -0.06456 | -0.01486 | -0.17608 | -0.16879 | -0.25687 |  | 0.971823 | 0.073237 | 0.05354 | 0.981114 | 0.412832 | 0.409316 |
| **SD** | 0.008062 | -0.05192 | 0.049153 | -0.26438 | -0.15771 | 0.077782 | -0.08282 | -0.07256 | -0.11502 | -0.15508 | -0.00956 | 0 | 0.440386 | 0.966117 | 0.312452 | 0.424111 | 0.657002 |
| **SA** | -0.00175 | -0.04462 | -0.0209 | -0.13209 | -0.01224 | -0.07984 | -0.04482 | 0.12598 | 0.088417 | 0.104296 | -0.2287 | 0.137146 |  | 0.000109 | 0.025137 | 0.163002 | 0.112395 |
| **VD** | 0.016053 | 0.130037 | 0.026284 | 0.237124 | 0.135976 | 0.037478 | 0.033402 | -0.14803 | -0.11334 | -0.19236 | 0.206171 | 0.051017 | -0.49524 | 0 | 0.283274 | 0.068118 | 0.708405 |
| **QY** | 0.013187 | -0.08156 | -0.02096 | -0.06931 | -0.06746 | -0.03746 | 0.048437 | -0.09582 | -0.03479 | 0.076408 | 0.024047 | 0.132699 | 0.324326 | -0.11766 |  | 0.910664 | 0.002842 |
| **fv.fm** | 0.053025 | 0.285611 | 0.015659 | -0.07545 | -0.06356 | 0.210945 | -0.02433 | -0.1379 | -0.10188 | 0.080049 | 0.140487 | 0.141006 | -0.08807 | 0.349079 | 0.207642 | 0 | 0.033516 |
| **NPQ** | 0.013604 | 0.255824 | -0.00831 | -0.04909 | -0.02565 | 0.253054 | 0.089908 | 0.074553 | 0.120883 | 0.169953 | -0.13735 | -0.02154 | -0.2129 | -0.0049 | -0.45574 | 0.455739 | 0 |

**Table S4**. Spearman’s correlations between pairs of phenotypes of green (G) ‘Salanova’ plants grown under well-watered (WW) regimes and low (L) VPD. P-values in the upper-right corner, rho values in the bottom-left corner.

| **TA** | **Y2G** | **Int** | **FW** | **DW** | **UE** | **PT** | **ST** | **LET** | **LT** | **IS** | **SD** | **SA** | **VD** | **QY** | **fv.fm** | **NPQ** |  |
| --- | --- | --- | --- | --- | --- | --- | --- | --- | --- | --- | --- | --- | --- | --- | --- | --- | --- |
| **TA** | 0 | 3.98E-14 | 4.67E-24 | 0.888955 | 0.811444 | 0.969466 | 0.782315 | 0.808894 | 0.78338 | 0.781584 | 0.980411 | 0.708281 | 0.765131 | 0.703799 | 0.62409 | 0.650276 | 0.589859 |
| **Y2G** | -0.914158515 |  | 2.38E-14 | 0.780706 | 0.830634 | 0.912269 | 0.542508 | 0.520783 | 0.501335 | 0.615618 | 0.953764 | 0.83128 | 0.836361 | 0.608534 | 0.650497 | 0.71946 | 0.597444 |
| **Int** | 0.982459262 | -0.905059391 | 0 | 0.930071 | 0.848629 | 0.958289 | 0.737493 | 0.770739 | 0.739445 | 0.730438 | 0.99301 | 0.745832 | 0.795679 | 0.66843 | 0.660023 | 0.642388 | 0.59166 |
| **FW** | -0.039611061 | -0.052356854 | -0.031807417 |  | 1.23E-07 | 0.028572 | 0.02168 | 4.92E-05 | 7.43E-05 | 0.016014 | 0.482683 | 0.022161 | 0.560937 | 0.272666 | 0.848416 | 0.886839 | 0.705894 |
| **DW** | -0.044855311 | -0.052371787 | -0.040033228 | 0.675294473 | 0 | 0.208837 | 0.009176 | 0.000356 | 0.000367 | 0.049001 | 0.447892 | 0.045603 | 0.750449 | 0.218914 | 0.809054 | 0.845574 | 0.499042 |
| **UE** | 0.00404515 | -0.015114521 | 0.003545696 | -0.281015848 | -0.013346689 | 0 | 0.66094 | 0.064705 | 0.081056 | 0.549225 | 0.813042 | 0.0518 | 0.502752 | 0.693197 | 0.99667 | 0.79457 | 0.975003 |
| **PT** | 0.014747134 | 0.067141734 | -0.001519818 | -0.147829416 | -0.208719651 | 0.008811583 | 0 | 0.002916 | 8.86E-05 | 0.136793 | 0.502498 | 0.288341 | 0.483441 | 0.131861 | 0.352836 | 0.718532 | 0.612074 |
| **ST** | 0.001180703 | 0.089596566 | -0.009551912 | -0.480890141 | -0.366799689 | 0.237283339 | 0.339591354 |  | 2.27E-11 | 0.036495 | 0.668388 | 0.119401 | 0.364957 | 0.324293 | 0.736121 | 0.76491 | 0.900271 |
| **LET** | 0.001025531 | 0.091364386 | -0.006797554 | -0.452492991 | -0.324182211 | 0.260599306 | 0.636578486 | 0.904665702 | 0 | 0.025718 | 0.9087 | 0.086171 | 0.338302 | 0.230221 | 0.594678 | 0.694709 | 0.764307 |
| **LT** | -0.004555024 | 0.044135965 | -0.029250017 | -0.264322671 | -0.11600263 | 0.027286952 | 0.148242804 | 0.246429349 | 0.310623003 |  | 0.378533 | 0.206089 | 0.960608 | 0.694473 | 0.70912 | 0.353304 | 0.614519 |
| **IS** | 0.010103073 | -0.022274764 | 0.006433172 | -0.113601977 | -0.071813757 | -0.016165356 | 0.164597317 | -0.10974426 | -0.021131735 | 0.139812238 | 0 | 0.47531 | 0.154643 | 0.842114 | 0.724176 | 0.78431 | 0.721469 |
| **SD** | -0.019039284 | 0.039219072 | -0.006999361 | 0.279362832 | 0.268578152 | -0.180084468 | 0.004805903 | -0.06169438 | -0.11001745 | -0.070646912 | -0.087279791 |  | 0.039601 | 0.833677 | 0.950706 | 0.983876 | 0.953893 |
| **SA** | 0.067049835 | -0.026695091 | 0.055666669 | -0.019659396 | 0.074114998 | 0.086120764 | 0.133786865 | 0.144064413 | 0.167875115 | 0.000514122 | -0.184314533 | -0.252174852 | 0 | 0.362044 | 0.463972 | 0.937208 | 0.481563 |
| **VD** | -0.018016976 | 0.039795678 | -0.032173833 | -0.11198323 | -0.092428954 | -0.060350568 | 0.21882872 | 0.109403187 | 0.188275351 | 0.045293413 | -0.004829834 | 0.068083549 | 0.160489301 |  | 0.431023 | 0.839098 | 0.627186 |
| **QY** | -0.034363624 | 0.049943692 | -0.015578348 | -0.0367915 | -0.009984654 | 0.036173388 | -0.053026349 | 0.006232539 | -0.006197377 | 0.008441264 | -0.023088374 | 0.023000221 | -0.043157919 | -0.048795739 | 0 | 0.204156 | 0.154396 |
| **fv.fm** | -0.025710153 | 0.011189307 | -0.036502246 | -0.029788547 | 0.013488138 | 0.004599335 | 0.039437604 | 0.015220766 | 0.018602643 | -0.076120025 | 0.009615442 | 0.044223875 | 0.078600663 | 0.088528178 | 0.199999136 | 0 | 0.188085 |
| **NPQ** | -0.036410781 | 0.049495233 | -0.041491203 | -0.043756615 | -0.086517695 | 0.036196962 | -0.002153185 | 0.019726177 | 0.005102487 | -0.028825901 | -0.028778978 | 0.032499918 | -0.045342097 | -0.019928568 | 0.200615448 | 0.200618714 | 0 |

**Table S5**. Spearman’s correlations between pairs of phenotypes of red (R) ‘Salanova’ plants grown under well-watered (WW) regimes and low (L) VPD. P-values in the upper-right corner, rho values in the bottom-left corner.

| **TA** | **Y2G** | **Int** | **FW** | **DW** | **UE** | **PT** | **ST** | **LET** | **LT** | **IS** | **SD** | **SA** | **VD** | **QY** | **fv.fm** | **NPQ** |  |
| --- | --- | --- | --- | --- | --- | --- | --- | --- | --- | --- | --- | --- | --- | --- | --- | --- | --- |
| **TA** | 0 | 1.79E-08 | 1.56E-18 | 0.75222 | 0.505228 | 0.488186 | 0.926057 | 0.637326 | 0.678765 | 0.555398 | 0.545927 | 0.674686 | 0.416624 | 0.713636 | 0.864084 | 0.472339 | 0.787759 |
| **Y2G** | 0.777713 |  | 4.46E-08 | 0.996555 | 0.895818 | 0.43568 | 0.654839 | 0.898796 | 0.946858 | 0.2852 | 0.802432 | 0.566163 | 0.491864 | 0.284539 | 0.85074 | 0.48693 | 0.856191 |
| **Int** | 0.979434 | 0.748647 | 0 | 0.816425 | 0.4827 | 0.520787 | 0.87796 | 0.671593 | 0.663084 | 0.593611 | 0.533133 | 0.775657 | 0.301006 | 0.67472 | 0.863453 | 0.483805 | 0.799747 |
| **FW** | 0.008382 | 0.089631 | 0.038672 |  | 0.000198 | 0.619515 | 0.093451 | 0.986405 | 0.076055 | 0.180326 | 0.253585 | 0.000221 | 0.74471 | 0.02735 | 0.750772 | 0.460557 | 0.626716 |
| **DW** | 0.01542 | 0.142474 | 0.009092 | 0.592527 | 0 | 0.822278 | 0.511723 | 0.560545 | 0.515363 | 0.158888 | 0.259233 | 0.087649 | 0.038535 | 0.129017 | 0.749565 | 0.426217 | 0.679237 |
| **UE** | -0.00639 | -0.00715 | 0.018671 | -0.00183 | 0.07278 |  | 0.570439 | 0.786486 | 0.81612 | 0.080467 | 0.568234 | 0.697006 | 0.868412 | 0.826126 | 0.764866 | 0.797445 | 0.70401 |
| **PT** | 0.003307 | 0.119674 | -0.00606 | -0.18282 | -0.00827 | -0.09536 | 0 | 0.456821 | 5.88E-07 | 0.570421 | 0.965878 | 0.004637 | 0.749093 | 0.515516 | 0.807077 | 0.848961 | 0.869232 |
| **ST** | 0.029834 | 0.093869 | 0.044383 | 0.076969 | 0.215469 | 0.098066 | 0.029088 |  | 0.031809 | 0.627236 | 0.227489 | 0.75375 | 0.462387 | 0.66121 | 0.538425 | 0.313364 | 0.588927 |
| **LET** | 0.02167 | 0.110162 | 0.029937 | -0.16091 | 0.087316 | 0.138741 | 0.812207 | 0.461031 | 0 | 0.610341 | 0.869043 | 0.008127 | 0.475835 | 0.660484 | 0.686781 | 0.68726 | 0.977864 |
| **LT** | 0.015023 | -0.07446 | 0.020158 | -0.10206 | -0.05881 | 0.273823 | -0.12268 | 0.132748 | 0.207225 |  | 0.124009 | 0.630565 | 0.236505 | 0.107312 | 0.89079 | 0.736953 | 0.812776 |
| **IS** | -0.00132 | 0.041767 | -0.00454 | 0.143635 | 0.148078 | -0.008 | 0.028803 | 0.243111 | 0.120077 | -0.12299 | 0 | 0.245204 | 0.422897 | 0.029987 | 0.528564 | 0.510101 | 0.596499 |
| **SD** | -0.00947 | -0.07884 | 0.032775 | 0.508505 | 0.241304 | -0.01894 | -0.37718 | 0.011881 | -0.28834 | 0.025414 | 0.197 |  | 0.658868 | 0.118516 | 0.659222 | 0.624237 | 0.525143 |
| **SA** | 0.004947 | 0.003211 | -0.06905 | 0.040257 | 0.4301 | 0.102996 | -0.00577 | -0.0512 | -0.03375 | -0.09955 | 0.193999 | -0.09973 | 0 | 0.653495 | 0.943206 | 0.963054 | 0.999647 |
| **VD** | 0.002282 | -0.15354 | 0.000309 | -0.27294 | -0.11243 | 0.004717 | -0.13471 | 0.01743 | -0.06038 | 0.224135 | -0.2463 | -0.20513 | 0.136939 |  | 0.821382 | 0.786015 | 0.952214 |
| **QY** | 0.023829 | 0.020322 | 0.023177 | 0.004374 | 0.021232 | 0.06031 | -0.02383 | -0.01648 | -0.02441 | 0.036687 | -0.03442 | -0.03289 | 0.044103 | 0.039485 | 0 | 0.025312 | 1.10E-05 |
| **fv.fm** | -0.08953 | -0.0542 | -0.07579 | -0.08918 | -0.07913 | 0.027645 | -0.0304 | -0.1068 | -0.04902 | 0.049059 | -0.05024 | -0.03721 | 0.028624 | 0.013394 | 0.201241 |  | 1.88E-05 |
| **NPQ** | 0.037167 | 0.01695 | 0.033021 | 0.046081 | 0.03633 | -0.03554 | -0.02911 | 0.058266 | -0.00375 | -0.00852 | 0.056945 | 0.097775 | 0.021657 | 0.028908 | -0.60124 | -0.60062 | 0 |

**Table S6**. Spearman’s correlations between pairs of phenotypes of green (G) ‘Salanova’ plants grown under low-watered (LW) regimes and high (H) VPD. P-values in the upper-right corner, rho values in the bottom-left corner.

| **TA** | **Y2G** | **Int** | **FW** | **DW** | **UE** | **PT** | **ST** | **LET** | **LT** | **IS** | **SD** | **SA** | **VD** | **QY** | **fv.fm** | **NPQ** |  |
| --- | --- | --- | --- | --- | --- | --- | --- | --- | --- | --- | --- | --- | --- | --- | --- | --- | --- |
| **TA** | 0 | 0.018832 | 4.07E-10 | 0.506444 | 0.726987 | 0.972942 | 0.955823 | 0.933495 | 0.966101 | 0.801213 | 0.867657 | 0.70999 | 0.921978 | 0.423519 | 0.405457 | 0.543036 | 0.414107 |
| **Y2G** | -0.33629 |  | 0.009707 | 0.03051 | 0.089556 | 0.77264 | 0.519723 | 0.128049 | 0.403907 | 0.40185 | 0.830122 | 0.27119 | 0.626534 | 0.515586 | 0.406181 | 0.010591 | 0.312811 |
| **Int** | 0.792635 | -0.3554 | 0 | 0.578132 | 0.714096 | 0.839456 | 0.922732 | 0.902198 | 0.709298 | 0.837716 | 0.895211 | 0.864101 | 0.736571 | 0.392971 | 0.391589 | 0.749544 | 0.414683 |
| **FW** | 0.22277 | 0.413523 | 0.211946 |  | 3.51E-08 | 0.012734 | 0.26397 | 0.236108 | 0.976681 | 0.739127 | 0.899935 | 0.022514 | 0.628528 | 0.369803 | 0.001148 | 4.92E-08 | 0.000437 |
| **DW** | 0.12704 | 0.283909 | 0.134292 | 0.841206 | 0 | 0.000122 | 0.138574 | 0.241462 | 0.653863 | 0.747818 | 0.359222 | 0.030289 | 0.793123 | 0.48278 | 0.00316 | 0.000205 | 0.001583 |
| **UE** | -0.01717 | -0.02446 | 0.046642 | 0.393815 | 0.663795 | 0 | 0.316593 | 0.762564 | 0.113082 | 0.963572 | 0.121346 | 0.045906 | 0.638827 | 0.699801 | 0.0386 | 0.182964 | 0.034831 |
| **PT** | 0.061468 | 0.084433 | 0.063087 | 0.252221 | 0.330098 | 0.221941 | 0 | 0.004009 | 0.377569 | 0.341101 | 0.887229 | 0.444109 | 0.328832 | 0.248299 | 0.692127 | 0.388615 | 0.797433 |
| **ST** | 0.111797 | 0.258379 | 0.049721 | 0.269042 | 0.299008 | -0.07762 | 0.457876 |  | 0.059522 | 0.054913 | 0.803872 | 0.754418 | 0.090498 | 0.008497 | 0.645936 | 0.17674 | 0.779311 |
| **LET** | 0.071601 | 0.150963 | 0.004261 | 0.131587 | 0.040472 | -0.15121 | 0.130151 | 0.298192 | 0 | 0.994509 | 0.033311 | 0.656714 | 0.652313 | 0.150847 | 0.212877 | 0.548454 | 0.22398 |
| **LT** | 0.042041 | 0.165827 | 0.047243 | 0.165058 | 0.10491 | 0.062512 | 0.144127 | 0.291442 | -0.00916 |  | 0.273169 | 0.797098 | 0.194379 | 0.069964 | 0.507967 | 0.832272 | 0.589156 |
| **IS** | 0.006471 | 0.008135 | 0.062119 | 0.044427 | 0.21643 | 0.225762 | -0.02574 | 0.108939 | -0.26632 | 0.174945 | 0 | 0.40057 | 0.177743 | 0.784793 | 0.924701 | 0.685103 | 0.846416 |
| **SD** | -0.04863 | -0.08912 | 0.006306 | -0.19604 | -0.1879 | -0.26756 | -0.06249 | -0.01933 | 0.062623 | -0.01748 | -0.12685 |  | 0.070714 | 0.707022 | 0.247824 | 0.009627 | 0.156682 |
| **SA** | 0.009341 | 0.081842 | -0.0299 | 0.099167 | -0.09271 | -0.13857 | -0.04851 | -0.21624 | -0.02658 | -0.10045 | -0.17227 | -0.29082 | 0 | 0.090258 | 0.284832 | 0.249091 | 0.236483 |
| **VD** | -0.05261 | -0.04681 | -0.09357 | -0.03953 | 0.001143 | 0.132333 | -0.06522 | -0.25924 | -0.15882 | -0.17923 | -0.01634 | -0.03586 | 0.263183 |  | 0.696996 | 0.39335 | 0.745712 |
| **QY** | 0.11669 | 0.08419 | 0.119348 | 0.41977 | 0.381553 | 0.263374 | -0.09784 | -0.09491 | -0.18728 | -0.10415 | -0.02295 | -0.04196 | 0.145269 | -0.1019 | 0 | 0.005767 | 4.35E-16 |
| **fv.fm** | 0.199007 | 0.436323 | 0.114092 | 0.781652 | 0.597479 | 0.091378 | 0.158118 | 0.291137 | 0.210038 | 0.099684 | -0.05574 | -0.34122 | 0.212734 | -0.04578 | 0.352444 |  | 0.002239 |
| **NPQ** | -0.12042 | -0.12767 | -0.12548 | -0.44899 | -0.42258 | -0.24972 | 0.064742 | 0.033744 | 0.184104 | -0.02004 | 0.11309 | 0.113095 | -0.18353 | 0.050611 | -0.94428 | -0.41326 | 0 |

**Table S7**. Spearman’s correlations between pairs of phenotypes of red (R) ‘Salanova’ plants grown under low-watered (LW) regimes and high (H) VPD. P-values in the upper-right corner, rho values in the bottom-left corner.

| **TA** | **Y2G** | **Int** | **FW** | **DW** | **UE** | **PT** | **ST** | **LET** | **LT** | **IS** | **SD** | **SA** | **VD** | **QY** | **fv.fm** | **NPQ** |  |
| --- | --- | --- | --- | --- | --- | --- | --- | --- | --- | --- | --- | --- | --- | --- | --- | --- | --- |
| **TA** | 0 | 0.079722 | 4.46E-10 | 0.799205 | 0.4819 | 0.893436 | 0.680136 | 0.868608 | 0.776498 | 0.271875 | 0.369887 | 0.367814 | 0.295613 | 0.593858 | 0.165011 | 0.781695 | 0.427128 |
| **Y2G** | -0.28404935 |  | 0.164501 | 0.094227 | 0.195988 | 0.267151 | 0.020451 | 0.993188 | 0.130102 | 0.786829 | 0.504773 | 0.180649 | 0.186292 | 0.64631 | 0.527429 | 0.030406 | 0.151922 |
| **Int** | 0.850149283 | -0.103840651 | 0 | 0.888663 | 0.762703 | 0.948765 | 0.629564 | 0.700107 | 0.622383 | 0.451294 | 0.283762 | 0.550086 | 0.516644 | 0.647742 | 0.418555 | 0.959701 | 0.773259 |
| **FW** | 0.039445919 | 0.21049398 | -0.003807238 |  | 1.38E-11 | 3.96E-05 | 0.060119 | 0.679581 | 0.727615 | 0.243148 | 0.665322 | 0.00071 | 1.47E-05 | 0.431236 | 0.002315 | 0.000172 | 0.00019 |
| **DW** | 0.163978949 | 0.148780405 | 0.107192114 | 0.858690047 | 0 | 1.21E-05 | 0.13748 | 0.789062 | 0.819826 | 0.20656 | 0.55215 | 0.000773 | 1.04E-05 | 0.45221 | 0.000836 | 0.000791 | 0.00026 |
| **UE** | 0.05366703 | 0.164967855 | 0.061122774 | 0.672435815 | 0.783514645 |  | 0.22673 | 0.822141 | 0.685753 | 0.348904 | 0.397894 | 0.204076 | 0.032961 | 0.244726 | 0.123488 | 0.155002 | 0.110159 |
| **PT** | 0.070231307 | -0.231469785 | 0.102595321 | -0.179255175 | -0.072550756 | -0.064236478 | 0 | 0.200188 | 0.281165 | 0.963062 | 0.748005 | 0.173484 | 0.196626 | 0.898572 | 0.588516 | 0.014554 | 0.083178 |
| **ST** | 0.040043168 | 0.047758045 | -0.006840221 | 0.160251321 | 0.089412411 | 0.083130479 | 0.251966732 |  | 0.519926 | 0.473634 | 0.194422 | 0.970775 | 0.654036 | 0.436695 | 0.611271 | 0.915513 | 0.929961 |
| **LET** | -0.002258342 | -0.16619893 | -0.041775152 | 0.056838723 | 0.085587893 | 0.128887131 | 0.127598571 | 0.126055681 | 0 | 0.961992 | 0.935714 | 0.110489 | 0.65678 | 0.875227 | 0.783091 | 0.082468 | 0.335885 |
| **LT** | -0.064014541 | 0.023901229 | 0.017948915 | -0.024176836 | -0.019104661 | 0.020463144 | 0.023326851 | 0.005941103 | 0.053599397 |  | 0.25394 | 0.582026 | 0.261419 | 0.759096 | 0.058467 | 0.51027 | 0.150072 |
| **IS** | -0.083054029 | -0.104929482 | -0.125507836 | -0.01337711 | -0.039698219 | -0.083227717 | -0.018293324 | -0.12306766 | 0.022039145 | 0.172420224 | 0 | 0.850562 | 0.993805 | 0.310867 | 0.500648 | 0.996573 | 0.738597 |
| **SD** | 0.168655196 | 0.149678023 | 0.15863397 | 0.445417075 | 0.504635236 | 0.064194535 | -0.003619548 | 0.032780013 | -0.196116174 | 0.109198501 | 0.10134897 |  | 7.29E-07 | 0.801102 | 0.002333 | 1.72E-07 | 4.45E-05 |
| **SA** | -0.158640267 | -0.135710002 | -0.117855642 | -0.526734184 | -0.532072867 | -0.206265114 | 0.090603068 | -0.074710618 | -0.047509633 | 0.1009574 | -0.075278389 | -0.608519265 | 0 | 0.984152 | 0.000191 | 8.75E-06 | 2.08E-05 |
| **VD** | -0.027864909 | 0.097894295 | -0.014323657 | -0.086288033 | -0.031195245 | -0.111468135 | 0.035719412 | 0.145338954 | 0.02298005 | 0.09599226 | -0.131295632 | 0.005207494 | -0.069567879 |  | 0.902116 | 0.903589 | 0.753015 |
| **QY** | -0.178170071 | 0.019962993 | -0.055125523 | -0.282486146 | -0.378931312 | -0.054492439 | -0.042103077 | -0.023705529 | 0.006073662 | 0.257057734 | 0.112554243 | -0.24174931 | 0.439289577 | 0.003664765 | 0 | 0.002675 | 1.53E-07 |
| **fv.fm** | 0.071018559 | 0.316952784 | 0.031475937 | 0.558095997 | 0.467968048 | 0.120974568 | -0.286029133 | 0.120511205 | -0.214294876 | 0.08527779 | 0.073620855 | 0.704495089 | -0.512407695 | 0.135915135 | -0.235601922 |  | 2.36E-06 |
| **NPQ** | 0.12475915 | 0.158528606 | 0.025020104 | 0.475924945 | 0.458024042 | 0.115048078 | -0.194019624 | 0.011913709 | -0.071314543 | -0.121752269 | -0.023804491 | 0.486124908 | -0.440793459 | -0.036139086 | -0.73374027 | 0.657347299 | 0 |

**Table S8.** Spearman’s correlations between pairs of phenotypes of green (G) ‘Salanova’ plants grown under well-watered (WW) regimes and high (H) VPD. P-values in the upper-right corner, rho values in the bottom-left corner.

| **TA** | **Y2G** | **Int** | **FW** | **DW** | **UE** | **PT** | **ST** | **LET** | **LT** | **IS** | **SD** | **SA** | **VD** | **QY** | **fv.fm** | **NPQ** |  |
| --- | --- | --- | --- | --- | --- | --- | --- | --- | --- | --- | --- | --- | --- | --- | --- | --- | --- |
| **TA** | 0 | 5.60E-11 | 1.35E-19 | 0.689111 | 0.548175 | 0.984863 | 0.9128 | 0.936655 | 0.983591 | 0.970312 | 0.690441 | 0.570125 | 0.718816 | 0.943124 | 0.714522 | 0.777398 | 0.83945 |
| **Y2G** | -0.28404935 |  | 5.82E-12 | 0.809203 | 0.970196 | 0.866824 | 0.827324 | 0.428761 | 0.50153 | 0.814539 | 0.583102 | 0.590978 | 0.627055 | 0.922423 | 0.947571 | 0.858725 | 0.746037 |
| **Int** | 0.850149283 | -0.103840651 | 0 | 0.832296 | 0.669151 | 0.945243 | 0.998806 | 0.835973 | 0.871023 | 0.908608 | 0.704036 | 0.557516 | 0.687331 | 0.974786 | 0.823022 | 0.860983 | 0.991458 |
| **FW** | 0.039445919 | 0.21049398 | -0.003807238 |  | 9.92E-11 | 0.039648 | 0.256601 | 0.019114 | 0.002029 | 0.898752 | 0.387738 | 0.738502 | 0.906581 | 0.564115 | 0.072255 | 0.010708 | 3.80E-05 |
| **DW** | 0.163978949 | 0.148780405 | 0.107192114 | 0.858690047 | 0 | 0.034977 | 0.389176 | 0.032905 | 0.004697 | 0.928447 | 0.279763 | 0.497941 | 0.911868 | 0.79483 | 0.102264 | 0.008078 | 7.14E-05 |
| **UE** | 0.05366703 | 0.164967855 | 0.061122774 | 0.672435815 | 0.783514645 |  | 0.51275 | 0.279901 | 0.054483 | 0.923058 | 0.433435 | 0.377264 | 0.869174 | 0.059377 | 0.091822 | 0.511944 | 0.004567 |
| **PT** | 0.070231307 | -0.231469785 | 0.102595321 | -0.179255175 | -0.072550756 | -0.064236478 | 0 | 0.158976 | 0.192555 | 0.197525 | 0.625861 | 0.921191 | 0.548984 | 0.315837 | 0.05001 | 0.566749 | 0.086918 |
| **ST** | 0.040043168 | 0.047758045 | -0.006840221 | 0.160251321 | 0.089412411 | 0.083130479 | 0.251966732 |  | 4.47E-07 | 0.336746 | 0.268665 | 0.278837 | 0.294577 | 0.874121 | 0.03044 | 0.075811 | 0.021027 |
| **LET** | -0.002258342 | -0.16619893 | -0.041775152 | 0.056838723 | 0.085587893 | 0.128887131 | 0.127598571 | 0.126055681 | 0 | 0.501307 | 0.856004 | 0.290485 | 0.359135 | 0.981982 | 0.035986 | 0.036312 | 0.002893 |
| **LT** | -0.064014541 | 0.023901229 | 0.017948915 | -0.024176836 | -0.019104661 | 0.020463144 | 0.023326851 | 0.005941103 | 0.053599397 |  | 0.702018 | 0.722651 | 0.851522 | 0.509866 | 0.368168 | 0.346094 | 0.384584 |
| **IS** | -0.083054029 | -0.104929482 | -0.125507836 | -0.01337711 | -0.039698219 | -0.083227717 | -0.018293324 | -0.12306766 | 0.022039145 | 0.172420224 | 0 | 0.008155 | 0.444241 | 0.442275 | 0.48954 | 0.4051 | 0.490389 |
| **SD** | 0.168655196 | 0.149678023 | 0.15863397 | 0.445417075 | 0.504635236 | 0.064194535 | -0.003619548 | 0.032780013 | -0.196116174 | 0.109198501 | 0.10134897 |  | 0.024927 | 0.030484 | 0.287642 | 0.189006 | 0.906432 |
| **SA** | -0.158640267 | -0.135710002 | -0.117855642 | -0.526734184 | -0.532072867 | -0.206265114 | 0.090603068 | -0.074710618 | -0.047509633 | 0.1009574 | -0.075278389 | -0.608519265 | 0 | 0.957495 | 0.880637 | 0.8467 | 0.684258 |
| **VD** | -0.027864909 | 0.097894295 | -0.014323657 | -0.086288033 | -0.031195245 | -0.111468135 | 0.035719412 | 0.145338954 | 0.02298005 | 0.09599226 | -0.131295632 | 0.005207494 | -0.069567879 |  | 0.257017 | 0.014869 | 0.976261 |
| **QY** | -0.178170071 | 0.019962993 | -0.055125523 | -0.282486146 | -0.378931312 | -0.054492439 | -0.042103077 | -0.023705529 | 0.006073662 | 0.257057734 | 0.112554243 | -0.24174931 | 0.439289577 | 0.003664765 | 0 | 0.804983 | 0.001602 |
| **fv.fm** | 0.071018559 | 0.316952784 | 0.031475937 | 0.558095997 | 0.467968048 | 0.120974568 | -0.286029133 | 0.120511205 | -0.214294876 | 0.08527779 | 0.073620855 | 0.704495089 | -0.512407695 | 0.135915135 | -0.235601922 |  | 0.022472 |
| **NPQ** | 0.12475915 | 0.158528606 | 0.025020104 | 0.475924945 | 0.458024042 | 0.115048078 | -0.194019624 | 0.011913709 | -0.071314543 | -0.121752269 | -0.023804491 | 0.486124908 | -0.440793459 | -0.036139086 | -0.73374027 | 0.657347299 | 0 |

**Table S9.** Spearman’s correlations between pairs of phenotypes of red (R) ‘Salanova’ plants grown under well-watered (WW) regimes and high (H) VPD. P-values in the upper-right corner, rho values in the bottom-left corner.

| **TA** | **Y2G** | **Int** | **FW** | **DW** | **UE** | **PT** | **ST** | **LET** | **LT** | **IS** | **SD** | **SA** | **VD** | **QY** | **fv.fm** | **NPQ** |  |
| --- | --- | --- | --- | --- | --- | --- | --- | --- | --- | --- | --- | --- | --- | --- | --- | --- | --- |
| **TA** | 0 | 3.04E-07 | 1.39E-15 | 0.829159 | 0.367305 | 0.734057 | 0.591535 | 0.823315 | 0.719938 | 0.56491 | 0.756638 | 0.933654 | 0.735465 | 0.964906 | 0.73932 | 0.751196 | 0.912775 |
| **Y2G** | 0.708543248 |  | 4.02E-07 | 0.907103 | 0.101148 | 0.272443 | 0.305682 | 0.690669 | 0.804961 | 0.492103 | 0.90371 | 0.497753 | 0.760799 | 0.832001 | 0.769864 | 0.987861 | 0.855814 |
| **Int** | 0.978331745 | 0.7038354 | 0 | 0.904273 | 0.365974 | 0.686827 | 0.499431 | 0.760296 | 0.643409 | 0.432543 | 0.607564 | 0.858107 | 0.631681 | 0.744667 | 0.95505 | 0.952513 | 0.810182 |
| **FW** | 0.096375901 | 0.045353871 | 0.052067186 |  | 0.028868 | 0.126839 | 0.169891 | 0.025672 | 0.084816 | 0.225317 | 0.459696 | 0.944753 | 0.459254 | 0.178511 | 0.686927 | 0.917997 | 0.265318 |
| **DW** | -0.091314832 | -0.201105106 | -0.107030007 | 0.33394784 | 0 | 0.00435 | 0.850774 | 0.000482 | 0.001991 | 0.549731 | 0.616948 | 0.02335 | 0.159504 | 0.43821 | 0.489169 | 0.693994 | 0.234684 |
| **UE** | -0.000598447 | -0.096251512 | -0.00368347 | 0.215496461 | 0.331730213 |  | 0.42131 | 0.003153 | 0.045826 | 0.219009 | 0.376505 | 0.011974 | 0.644955 | 0.244365 | 0.76463 | 0.22431 | 0.967387 |
| **PT** | 0.01887389 | -0.10062467 | 0.000601512 | -0.155693485 | 0.099178471 | 0.122660442 | 0 | 0.254832 | 0.209569 | 0.038096 | 0.990647 | 0.047358 | 0.483 | 0.361104 | 0.115957 | 0.029518 | 0.07026 |
| **ST** | 0.017044293 | 0.1129728 | 0.003293328 | -0.188428256 | -0.42347843 | -0.407387638 | 0.235800995 |  | 8.71E-08 | 0.830434 | 0.215007 | 0.234455 | 0.37962 | 0.90619 | 0.268816 | 0.387314 | 0.096438 |
| **LET** | 0.010428802 | 0.121921762 | -0.008187598 | -0.090191895 | -0.350226769 | -0.155401373 | 0.215740829 | 0.733343452 | 0 | 0.648625 | 0.455352 | 0.290198 | 0.283703 | 0.774983 | 0.26734 | 0.256174 | 0.109869 |
| **LT** | 0.016297111 | 0.025573923 | -0.013352846 | -0.157282152 | 0.170917464 | 0.214821885 | 0.264918505 | 0.055839917 | 0.125189486 |  | 0.474356 | 0.502568 | 0.716859 | 0.159976 | 0.02614 | 0.031533 | 0.018732 |
| **IS** | 0.016788236 | 0.030035921 | -0.00871295 | -0.087703015 | 0.027042894 | -0.097126234 | -0.036412921 | 0.222337265 | 0.061953992 | 0.09127202 | 0 | 0.218714 | 0.369979 | 0.024732 | 0.05899 | 0.143407 | 0.068739 |
| **SD** | -0.008022092 | 0.118590446 | -0.035884532 | 0.081800574 | -0.319881994 | -0.318851887 | -0.306469767 | 0.118444018 | 0.128230438 | -0.019296302 | 0.155909375 |  | 0.245191 | 0.529082 | 0.47216 | 0.951468 | 0.520449 |
| **SA** | 0.008476984 | -0.01202721 | 0.008399625 | -0.042058836 | -0.167006612 | -0.016021076 | 0.100856843 | 0.06955304 | 0.137327542 | 0.019990915 | 0.076072242 | 0.159314812 | 0 | 0.106623 | 0.043546 | 0.058031 | 0.02693 |
| **VD** | 0.009732619 | 0.039193781 | 0.078174612 | -0.25050776 | -0.178722888 | -0.124878993 | -0.06699841 | 0.09852691 | -0.028712273 | -0.08731302 | -0.30398021 | -0.129525134 | -0.167616134 |  | 0.002413 | 0.000417 | 0.018065 |
| **QY** | -0.039768333 | -0.040864588 | 0.038137544 | 0.031345987 | 0.077191285 | 0.002135346 | -0.139853872 | -0.089146904 | -0.050415701 | -0.222310407 | -0.182747 | -0.112562096 | -0.174552449 | 0.394992717 | 0 | 5.62E-07 | 6.49E-11 |
| **fv.fm** | -0.009199304 | 0.106445659 | 0.059852857 | 0.030065543 | 0.143879275 | -0.222826079 | -0.207810497 | -0.049196853 | -0.06145501 | -0.115042964 | -0.059016871 | 0.104626622 | -0.119851532 | 0.52955158 | 0.67545045 |  | 7.57E-06 |
| **NPQ** | 0.058407328 | 0.083699736 | -0.009913355 | -0.130438408 | -0.148103134 | 0.025787754 | 0.230266626 | 0.269791283 | 0.201193114 | 0.332708104 | 0.225121254 | 0.089985555 | 0.293271619 | -0.217376566 | -0.822011595 | -0.499635994 | 0 |
